# Exploring the Potential Application of Matrimid^®^ and ZIFs-Based Membranes for Hydrogen Recovery: A Review

**DOI:** 10.3390/polym13081292

**Published:** 2021-04-15

**Authors:** Pablo Fernández-Castro, Alfredo Ortiz, Daniel Gorri

**Affiliations:** Departamento de Ingenierías Química y Biomolecular, ETSIIyT, Universidad de Cantabria, Avda. de Los Castros s/n, 39005 Santander, Spain; fcastrop@unican.es (P.F.-C.); alfredo.ortizsainz@unican.es (A.O.)

**Keywords:** Matrimid, mixed matrix membranes, zeolitic imidazolate framework, hydrogen recovery, gas separation

## Abstract

Hydrogen recovery is at the center of the energy transition guidelines promoted by governments, owing to its applicability as an energy resource, but calls for energetically nonintensive recovery methods. The employment of polymeric membranes in selective gas separations has arisen as a potential alternative, as its established commercial availability demonstrates. However, enhanced features need to be developed to achieve adequate mechanical properties and the membrane performance that allows the obtention of hydrogen with the required industrial purity. Matrimid^®^, as a polyimide, is an attractive material providing relatively good performance to selectively recover hydrogen. As a consequence, this review aims to study and summarize the main results, mechanisms involved and advances in the use of Matrimid^®^ as a selective material for hydrogen separation to date, delving into membrane fabrication procedures that increase the effectiveness of hydrogen recovery, i.e., the addition of fillers (within which ZIFs have acquired extraordinary importance), chemical crosslinking or polymeric blending, among others.

## 1. Introduction

Hydrogen has gained remarkable attention in recent decades owing to its strong potential role in the development of clean and efficient energy, e.g., the large amount of fuel cell studies demonstrate [1]. Its advance will diminish not only the fossil fuel consumption in transport, industry and buildings but also the release of pollutants into the environment, among which greenhouse gases are highlighted [2]. For instance, the European plan for the decarbonization process, signed and ratified in the Paris agreement, will require hydrogen to achieve the energy transition, involving the decarbonization of (i) the gas grid that supplies energy in industry and households; (ii) the public, private and commercial transport; and (iii) the industrial high-grade heat production or the use of this fuel as raw material [3]. As a consequence of European policies in terms of the decarbonization process, the sector coupling (SC) concept has arisen owing to the decarbonization potential of renewable power and the integrated optimization of the whole energy system by merging renewable energies with consumption sectors: industry, mobility, residential and service sectors [4,5]. The potential applications of SC are power electrification (substituting fossil fuel applications and technologies), power-to-gas/liquids (electrolysis of water to obtain hydrogen that can be converted into methane or methanol) and power-to-heat. SC in multienergy systems, such as the industrial sector, could render processes environmentally friendly, reducing prices and increasing the competitivity. For instance, in ammonia and methanol manufacture industries, renewable energy could be used for electricity generation (used in the production process and providing electricity to community), for hydrogen production by water electrolysis and for hydrogen recovery after the production process [6,7].

According to the Fuel Cells and Hydrogen Joint Undertaking (FCH), formed by 17 companies and organizations, 2250 terawatt hours (TWh) of hydrogen could be generated in Europe in 2050 (one quarter of the EU’s total energy demand), causing a positive impact on CO_2_ emissions—a reduction of 560 Mt. This scenario implies the necessity of increasing hydrogen availability from primary and secondary resources, which depend on the regional availability of coal, natural gas, biomass, nuclear, solar, wind and electricity using electrolyzers, and at the same time calls for the recovery of hydrogen lost in industrial waste gas streams [8,9,10,11]. The major hydrogen-rich off gas streams include captive industries related to ammonia and methanol manufacture; oil refining; and by-product industries, e.g., petrochemical, steel-making and chloro-alkali industries [12,13]. This surplus hydrogen recovered from exhaust gas mixtures, such as ammonia purge gas, methanol purge gas or coke oven gas, may find application, therefore, as energy resources or raw materials for other chemical processes, such as ammonia, fertilizer and methanol production [14].

The best available technologies for hydrogen recovery from different waste streams include cryogenic separation, membrane technology and pressure swing adsorption (PSA) [15]. Commercial applications are nowadays mainly based on the use of cryogenic separation and PSA [16,17,18]. The main advantages of these technologies are their applicability to large industrial scale and the potential for achieving high hydrogen purity. Nevertheless, they have to deal with hydrogen losses, the use of a large amount of equipment or high energy requirements, among others. Consequently, membranes have arisen as an attractive and alternative technology for reaching high purities reducing the energy demand and, thus, providing a cost-effective method for hydrogen recovery. The fundamentals and general performance of membrane technology will not be covered in this review, and can be found elsewhere [19,20].

The research developed towards hydrogen recovery by means of membranes comprises the use of different materials, such as polymer, metallic, silica, zeolite and carbon-based membranes, whose main features are gathered in Table 1 [16,17,21,22,23]. The characteristic transport mechanism for each hydrogen-selective membrane is solution/diffusion for dense polymer, ceramic and metallic membranes; molecular sieving for microporous ceramic; and surface diffusion and molecular sieving for porous carbon [20].

**Figure 1:**
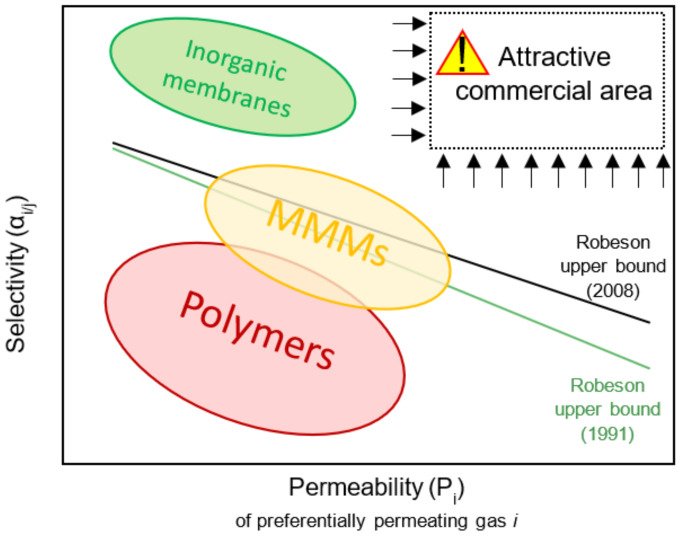
Relationship between permeability and selectivity. Current situation between different membrane composition regarding Robeson upper bound and the attractive commercial area. Adapted from Dechnik et al. [26].

Research on polymeric membranes has been extended during the last century for their implementation as viable technologies for hydrogen purification, focusing their goal on the performance comparison of different materials, their modification for reaching better features, or the addition of fillers that contribute to obtaining higher hydrogen permeability and selectivity [24,25]. In this sense, the combination of polymers with other materials, such as polymers, metals or zeolites, may overcome stability and sensitivity issues regarding aggressive media and increase hydrogen selectivity, leading to promising results in the application of polymeric membranes for gas separation (Figure 1) [26].

**Figure 2:**
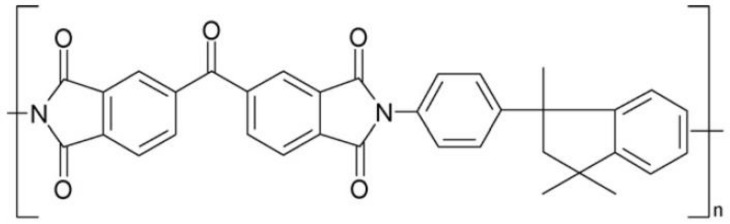
Chemical structure of Matrimid^®^ 5218.

Despite the availability of different research studies dealing with hydrogen recovery by commercial membranes already used for gas separation, such as PRISM^®^ (polysulfone), MEDAL^®^ (polyimide, polyamide), SEPAREX^®^ (cellulose acetate) or UBE^®^ (polyimide), which lead to H_2_/CO_2_ selectivity up to 3.8, currently, effort is focused on the search for materials that are more selective towards hydrogen [13,23]. PMDA-based (pyromellitic dianhydride), 6FDA-based (4,4′-(hexafluoroisopropylidene) diphthalic anhydride) polyimide membranes and Matrimid^®^ (5(6)-amino-1-(4′ aminophenyl)-1,3,-trimethylindane) constitute some of the polymers that are the most widely studied [27]. Among these polymers, Matrimid^®^ stands out due to its commercial availability, good chemical resistance and thermal properties (*T*_g_ ≈ 305–315 °C), high solubility in several solvents (e.g., dichloromethane-DCM-, tetrahydrofuran-THF- and *N*-methylpyrrolidone-NMP-) and good processability, and it exhibits a good tradeoff between H_2_ permeability and both H_2_/N_2_ and H_2_/CO_2_ selectivities, fulfilling the main features that should characterized membranes for ideal gas separation [28,29,30]. Matrimid^®^ 5218, whose chemical structure is represented in Figure 2, is obtained by the polycondensation of 3,3′,4,4′-benzophenone tetracarboxylic dianhydride (BTDA) and a mixture of two cycloaliphatic monomers, such as 5,6-amino-1-(40-aminophenyl) and 1,3,3-trimethylindane [31,32]. As glassy polymer, Matrimid^®^, and polyimides in general, is characterized by an excess of free volumes that contribute to the gas transport properties, conferring acceptable values of selectivity and permeability [25,28].

However, despite the relatively good performance of Matrimid^®^, better results in terms of permeability, selectivity and stability are needed. As a consequence, most research efforts and strategies associated with the improvement of Matrimid^®^ performance in hydrogen recovery are centered on the analysis of a Matrimid^®^ blend with other polymers, polymer modification or the addition of micro/nanosized fillers into the Matrimid^®^ polymer, leading the latter to the concept of mixed matrix membranes (MMMs) [27]. MMMs provide improved characteristics, exploiting the advantages of both components: permeability and selectivity of the inorganic fillers and cost and processing convenience of the polymer. Therefore, the selection of an appropriate polymer/filler pair, the assurance of obtaining good polymer/filler dispersions and the guarantee of obtaining adequate filler/polymer morphology are crucial in order to achieve optimal results [33]. Together with zeolites, zeolitic imidazolate frameworks (ZIFs), a subset of the metal organic frameworks (MOFs), have gained attention due to their compatibility and interaction with many polymers, creating a membrane structure that facilitates the flux of hydrogen preferentially over other mixture components, e.g., CH_4_ or CO_2_, usually referred to as the molecular sieving effect [26,34].

This review aims to contribute a further advance in the field of hydrogen recovery taking into the consideration the need for higher hydrogen production, the weaknesses of the current available processes used and the growing research interest in developing MMMs based on Matrimid^®^ and ZIF for gas separation processes. In this sense, details are provided which complement previously published information and results related to gas separation by means of MMMs, which have addressed gas separation from a general point of view without focusing on a particular material or in other gas mixtures out of the scope of this manuscript [24,25,27,30,33,35,36,37,38,39,40,41]. Hence, this manuscript gathers the main advances in hydrogen recovery through Matrimid^®^-based membranes, centering on the properties, permeation results, strengths and weaknesses revealed to date, and its comparison with other good performance polymeric membranes.

## 2. Pristine and Mixed Matrix Membranes Based on Matrimid^®^/ZIF

### 2.1. Membrane Preparation Techniques

#### 2.1.1. Flat Sheet Membranes

The procedures most commonly found in the literature are described below. First, commercial Matrimid^®^ polymer powder was commonly dried at ambient or vacuum pressure at temperatures above 110 °C overnight or for 24 h. Afterwards, this polymer was dissolved in different solvents, such as chloroform [42,43], NMP [44,45], DCM [31,46,47], dimethyl formamide (DMF) [48], 1,1,2,2-tetrachloroethane (TCE) [49], gamma-butyrolactone (GBL) and n-butanol [50]. Several polymer weight ratios have been tested with the aim of obtaining the best permeability and selectivity performances prior to the design of Matrimid^®^/ZIF mixtures. The next step consisted of the stirring of the solution until a homogeneous and clear solution was obtained.

Regarding ZIF fillers, they are commonly used as received from suppliers or synthesized from their raw materials [42,44]. When synthesized in the laboratory, they comprised mixture, stirring, sonication, centrifugation, washing and redispersion procedures. Prior to ZIF use, it was further sonicated in order to prevent particulate aggregation and degassing. ZIFs can also be used for the encapsulation of some nanoparticles (NPs), such as Pd, preventing their aggregation and improving the molecular sieving performance of ZIF [44]. For ZIF dispersion preparation, the same solvent as in the Matrimid^®^ solution was chosen.

Then, Matrimid^®^ and ZIF solutions were mixed. Once the ratio Matrimid^®^/ZIF was defined, the mixture of both solutions was mainly carried out following two procedures: (i) mixture with the desired amount of ZIF dispersion in one step; (ii) mixture of Matrimid^®^ solution and ZIF dispersion in several steps. In one-step mixtures, this was stirred overnight or for 24–48 h, and sonicated to remove the air bubbles and prepare a homogenous dispersion. In multistep mixtures, a sufficient amount of polymer, around 5–20 wt.% polymer solution, was added into the ZIF dispersion in a step called priming that prevents the agglomeration of particles [25,43]. This mixture was stirred and sonicated for 1–2 h, and further polymer solution was added to the mixture. This procedure was repeated until all the polymer solution was well mixed with the particle’s dispersion.

Next, the polymer solution was cast onto a clean glass disk, followed by a drying process at ambient conditions, applying temperature and/or vacuum or keeping the membrane under saturated solvent vapor. Once the membrane was removed from the glass disk, usually helped by distillated water, the membrane was further dried at ambient conditions or annealed at a high temperature and vacuum, targeting the removal of the solvent remaining inside the membrane.

#### 2.1.2. Hollow Fiber Membranes

In this section, the specific extrusion and co-extrusion conditions were not included as a consequence of the large number of solvents used, variety of flowrates selected, air gaps distances chosen, etc.; therefore, a general description of the main method employed for hollow fiber membrane (HFM) preparation is described. Among the possible HFM fabrication methods, such as thermally induced phase inversion, nonsolvent induced phase inversion or the electrospinning method, the second was preferably selected in the synthesis of Matrimid^®^-based membranes [51,52]. In particular, the dry-jet/wet-quench process was chosen, finding special application during co-extrusion through a triple-orifice spinneret.

**Figure 3:**
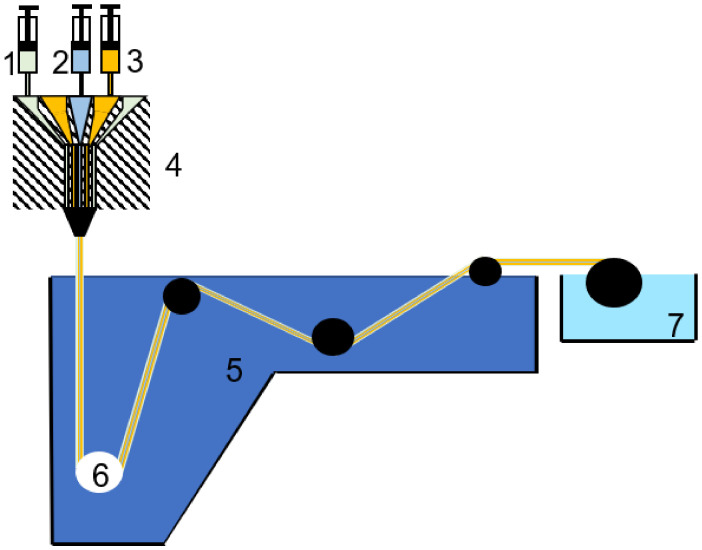
Dual-layer hollow fiber spinning by dry-jet/wet-quench set-up: (**1**) outer dope fluid, syringe pump and outer channel; (**2**) bore fluid, syringe pump and bore channel; (**3**) inner dope fluid, syringe pump and inner channel; (**4**) dual-layer spinneret; (**5**) coagulation bath; (**6**) fiber guiding wheel; (**7**) fiber collecting wheel.

During the dry-jet/wet-quench procedure, a dope solution, formed by the mixture of the polymer with solvents or nonsolvents, is spun through an air gap (dry-jet) into a coagulation bath (wet-quench). The stable skin layer is obtained in the air gap when volatile solvent and nonsolvents are evaporated, whereas the phase separation occurs in the coagulation bath. Owing to the local evaporation, close to the surface, a high polymer concentration is created, forming a dense top layer [53]. In co-extrusion processes, bore fluid, inner dope fluid and outer dope fluid (prepared as explained for the synthesis of flat sheet membranes) are simultaneously extruded and converge at the orifice of the spinneret (Figure 3) [51]. The nascent fiber is firstly put into contact with the bore fluid that acts as the coagulant, passes through an air gap and enters into the second coagulant (coagulant bath). The morphology of the membrane can be controlled by the spinneret design; the composition, temperature and flow rate of the bore fluid; the extrusion flow rate, pressure and temperature of the dopes and their viscosity; the air gap distance; the coagulation bath (coagulant and temperature selection); the elongational draw ratio and take-up speed, together with travelling distance; the drying process when removing solvents and nonsolvents from the HF; and the post-treatment and additional coating [54]. The post-treatment process includes not only solvent exchange and solvent removal but also physical aging to achieve the thermodynamic equilibrium of the fiber, its structure relaxation, free volume reduction and polymer chain packing density increase [55].

Some advantages attributed to dual-layer fibers over single-layer asymmetric fibers during MMM spinning are the reduction in material costs, the possibility of combining materials whose single employment is not feasible for industrial application, the porosity control, the obtention of cost-effective fibers and obtainment of higher fluxes [38].

### 2.2. Material and Thermo-Mechanical Characterization

A wide range of techniques has been used in order to correctly characterize Matrimid^®^ and Matrimid^®^/ZIF membranes and establish a relationship with the gas transport properties. In this section, the main characterization methods will be listed, and the main observations and results obtained will be related to permeation properties and discussed in Section 2.3. By means of scanning electron microscopy (SEM) and transmission electron microscopy (TEM), the cross-sectional morphology of the MMMs was analyzed; atomic force microscopy (AFM) allowed the surface characterization; X-ray diffraction (XRD) analyzed the structural properties and interchain spacing of the membrane, and the average *d*-spacing was obtained employing Bragg’s law; thermogravimetric analysis (TGA) and differential scanning calorimetry (DSC) were used for analyzing thermal stability and decomposition; a dynamic mechanical analyzer (DMA) enabled the investigation of temperature-dependent viscoelastic properties; Young’s modulus and hardness studies were carried out by nanoindentation experiments; the Instron universal machine allowed the measurement of stress–strain curves; fractional free volume (FFV) was calculated by the Bondi approach or by means of positron annihilation lifetime spectroscopy (PALS); energy dispersive X-ray (EDX) and X-ray photoelectron spectroscopy (XPS) were used for elemental analysis; gas sorption was measured by means of sorption cells and equipment; Fourier transform infrared spectroscopy (FTIR) and nuclear magnetic resonance (NMR) provided spectral information about chemical bonds for substance identification; through Brunauer–Emmett–Teller (BET) and Horvath–Kawazoe (HK) equations, the apparent surface area and pore size, respectively, were estimated [43,50,56,57,58,59].

### 2.3. Properties and Permeation Results of Pristine Matrimid^®^ Membrane

Throughout the manuscript, reported permeabilities and selectivities will be compared with the aim of studying the ability of the membrane to separate different gases, defining selectivity as *α_i,j_ = P_i_/P_j_*, where *P_i_* and *P_j_* are the permeabilities of components *i* and *j* [19]. Throughout the following sections, data from different pure gas permeation studies will be gathered in tables and depicted in figures, thus facilitating their interpretability; analyzed; and, finally, compared to binary and multicomponent gas mixtures if available.

#### 2.3.1. Flat Sheet Membranes

**Figure 4:**
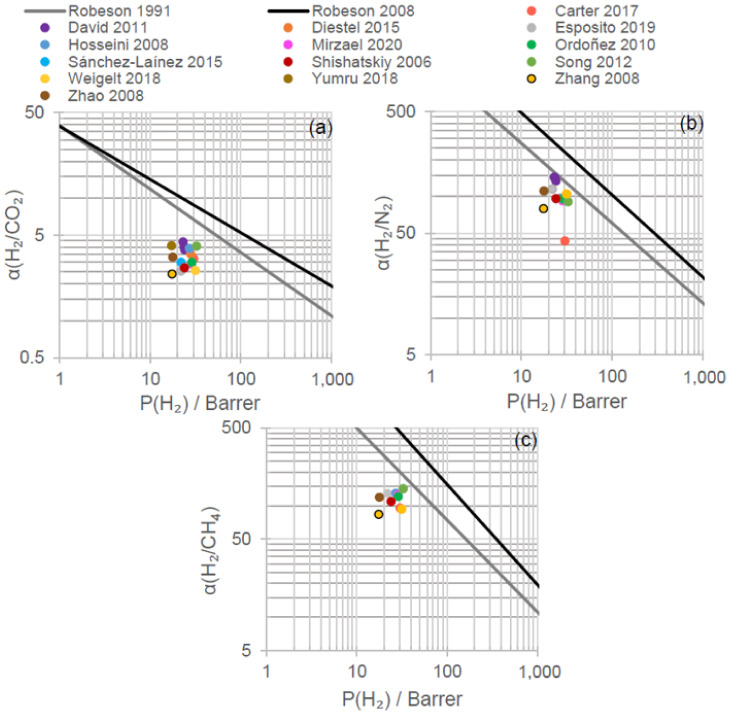
Robeson plot for (**a**) H_2_/CO_2_, (**b**) H_2_/N_2_ and (**c**) H_2_/CH_4_ single gas permeation tests through pristine Matrimid^®^ membranes. Reference correspondence is found at Table 2.

Pure Matrimid^®^ dense membranes have displayed selectivity towards hydrogen against other gases that can be found in exhaust streams, as is the case of nitrogen, carbon dioxide, carbon monoxide or methane. It is relevant to highlight that most of the research developed in hydrogen recovery by means of polymeric membranes, specifically by using Matrimid^®^, is focused on single gas or binary mixtures. In addition, most of the research is centered only on H_2_/N_2_ or H_2_/CO_2_ separation, whose main results in terms of single gas permeability and ideal selectivities are gathered in Figure 4 and further detailed in Table 2.

Figure 4 displays the comparison between pristine Matrimid^®^ membranes and the empirical upper bound (prior, 1991; present, 2008) relationships for polymeric membrane separation gases [60,61]. It shows that for H_2_/CO_2_, H_2_/N_2_ and H_2_/CH_4_, the permeability and selectivity values obtained in bibliography by using pristine Matrimid^®^ are lower than the state-of-the-art limits for polymeric gas membrane separation. During gas separation tests at near-ambient temperatures (15–30 °C), H_2_ single gas permeability ranged between 17 and 33 Barrer; N_2_, between 0.15 and 0.70 Barrer; and CO_2_, between 5.2 and 9.8 Barrer [31,42,43,44,45,46,47,48,49,50,58,62]. Consequently, the corresponding H_2_/N_2_ and H_2_/CO_2_ ideal selectivities were 80–144 and 3.0–4.4, respectively. To explain these differences, the kinetic diameters of the gases, their boiling points and their permeation mechanisms need to be considered.

**Table 2:** Main bibliographical data in terms of pure gas H_2_, N_2_, CO_2_ and CH_4_ permeabilities and selectivities through pristine Matrimid^®^ membrane.

Ref.	T (°C)	ΔP (Bar)	P_H_2__(Barrer)	P_N_2__(Barrer)	P_CO_2__(Barrer)	P_CH_4__(Barrer)	α_H_2_/N_2__	α_H_2_/CO_2__	α_H_2_/CH_4__	Solvent Used for Casting/Permeation Method
Carter (2017) [48]	35	3.0	30.3	0.70	9.5	0.32	43.2	3.2	94.6	DMF/time-lag
David (2011) [31,47]	30	2.0	24.1	0.18	6.4		133.9	3.8		DCM/continuous permeation with sweep gas
	30	4.0	23.7	0.17	5.7		138.8	4.0		
	30	6.0	23.1	0.16	5.2		144.4	4.4		
Diestel (2015) [46]	25	0.2	28.0		8.0			3.5		DCM/continuous permeation with sweep gas
Esposito (2019) [63]	25	1.0	21.9	0.19	8.6	115.3	2.5	0.2	128.8	DCM/time-lag
Hosseini (2008) [45]	35	H_2_: 3.5	27.2	0.28	7.0	0.21	97.0	3.9	129.3	NMP/time-lag
		Other gases: 10.0								
Mirzaei (2020) [44]	25	4.0	28.7	0.31	9.8	0.23	92.4	2.9	124.8	NMP/time-lag
Ordoñez (2010) [43]	35	1.7	28.9	0.31	9.5	0.24	95.1	3.0	120.4	chloroform/time-lag
Sánchez-Laínez (2015) [58]	35	2.0	22.0		7.3			3.0		chloroform/continuous permeation with sweep gas
	100		53.0		13.3			4.0		
	150		110.0		22.0			5.0		
	200		340.0		42.5			8.0		
Shishatskiy (2006) [50]	20–80	0.3	24.0	0.25	9.8	0.22	96.0	2.7	109.1	THF, NMP, G-BL, i-propanol, n-butanol, acetic acid and toluene/time-lag
Song (2012) [42]	22	4.0	32.7	0.36	8.1	0.23	90.9	4.1	142.1	chloroform/time-lag. Annealed at 230 °C
Weigelt 2018 [64]	30	1.0	31.6	0.3	12.3	105.3	2.6	0.3	92.9	chloroform/time-lag
Yumru (2018) [62]	35	4.0	17.3		4.2	0.30		4.1	66.5	NMP/time-lag
Zhang (2008) [49]	25	2.0	17.5	0.22	7.3	0.21	79.6	2.4	83.3	TCE/time-lag
Zhao (2008) [65]	35	2.0	17.8	0.16	8.9	0.15	110.9	3.3	118.7	THF/time-lag

In dense membranes, gas permeation is described by the solution-diffusion model whose driving force is the pressure or concentration gradient [19]. Hydrogen, nitrogen and carbon dioxide are characterized by different kinetic diameters—2.89, 3.64 and 3.30 Å, respectively—and boiling points—13.8, 77.4 and 194.7 K (CO_2_ sublimes), respectively [66,67]. As a consequence, hydrogen permeation is favored by diffusion selectivity due to its smaller diameter, whereas carbon dioxide permeation is favored by solubility selectivity owing to its higher condensability [39]. Glassy polymers, such as Matrimid^®^, are, then, hydrogen-selective as can be observed in Figure 4 and confirmed by SEM images that showed the absence of voids [48]. Regarding nitrogen, its higher kinetic diameter with respect to hydrogen or carbon dioxide together with its low boiling point led to lower N_2_ permeabilities and resulted in higher H_2_/N_2_ selectivity compared to H_2_/CO_2_.

In addition to N_2_ and CO_2_, although to a lower degree of significance, the permeability of other waste gas components, such as CH_4_ and CO, has been evaluated in several works. CH_4_ and CO are characterized by kinetic diameters of 3.80 and 3.76 Å, respectively, and boiling temperatures of 111.7 and 81.7 K [66,67]. In CH_4_ single gas permeation tests, permeabilities in the range of 0.21–0.32 Barrer were obtained, with a corresponding H_2_/CH_4_ selectivity of 66.5–142.7 (Figure 4) [42,43,44,45,50,62]. Meanwhile, in a CO single-gas permeation test, permeability reached 0.33 Barrer and H_2_/CO_2_ selectivity 54.8 [47]. The permeabilities obtained for CH_4_ and CO are consistent with those corresponding to N_2_ and CO_2_, due to the higher kinetic diameter of CH_4_ and CO, which hinders diffusion selectivity, and the relatively low boiling points compared to CO_2_ that do not favor solubility selectivity.

These outcomes were further confirmed by the diffusion and solubility coefficients estimated by the time-lag method. Diffusivity ratios for the gas pairs H_2_/CO_2_, H_2_/N_2_ and H_2_/CH_4_ of 379.3, 458.3 and 1970.0, respectively, and solubility ratios for CO_2_/H_2_, N_2_/H_2_ and CH_4_/H_2_ of 147.6, 5.2 and 18.1, respectively, were obtained [50]. From these data, the predominance of hydrogen diffusivity selectivity over carbon dioxide, nitrogen and methane can be inferred, especially regarding the latter. However, in terms of solubility selectivity, that corresponding to carbon dioxide is remarkable and justifies the overall selectivity of H_2_/CO_2_ where the diffusivity of hydrogen is counterbalanced by the solubility of carbon dioxide. Both gas pairs, H_2_/N_2_ and H_2_/CH_4_, showed similar overall selectivities that can be observed in the slight differences between diffusivity and solubility selectivity, obtaining higher diffusivity for nitrogen but higher solubility for methane. The slightly higher permeability of nitrogen compared to methane in polyimide membranes, such as Matrimid^®^, contrary to conventional glassy and rubbery polymers, is explained by the dominance of diffusivity selectivity, a consequence of the narrow gap sizes and the limited mobility of the polyimide chains [28].

It is noteworthy that most of the research available is focused on single gas permeation tests and, therefore, the interaction between gases has not been assessed, although there are several works that expand on this matter. In binary mixtures, a decrease in hydrogen (from 24.0 to 16.5 Barrer) and carbon dioxide (from 6.1 to 5.5) permeabilities was observed, especially for hydrogen, leading to a diminishment in H_2_/CO_2_ real selectivity (from 4.0 to 3.0) [31]. These changes are strongest when feed CO_2_ fugacity is increased, and this has been attributed by some studies in the literature to the competitive sorption phenomenon between hydrogen and carbon dioxide, owing to carbon dioxide displays higher affinity constant and solubility in glassy polymers, such as Matrimid^®^, which prevents hydrogen molecules from diffusing. On the other hand, Ordoñez et al. (2010) observed a slight decrease in real H_2_/CO_2_ (from 2.96 to 2.51) and CO_2_/CH_4_ (from 43.59 to 42.14) selectivities in feed gas mixtures composed by 50/50 mol.% H_2_/CO_2_ and 10/90 mol.% CO_2_/CH_4_, being almost negligible for the latter, which could be associated with standard deviations [43]. With regard to H_2_/N_2_ binary mixtures, at hydrogen concentrations higher than 10 vol.%, the polarization concentration was not found in the feed side, and hydrogen permeability leveled off to a constant value, displaying no competitive sorption between both gases [47]. No influence of carbon monoxide presence was observed in hydrogen permeability, and CO permeability remained constant with respect to the pure gas permeability [68].

Therefore, to date and to the best of our knowledge, investigations currently published have focused merely on a binary H_2_/CO_2_ mixture without assessing or comparing the real behavior of Matrimid^®^ recovery against ideal permeation measurements, complicating an appropriate comparison. Furthermore, it is important to highlight that despite the similarities in ideal selectivities gathered in Figure 4 and Table 2, the permeability of mixture gases through Matrimid^®^ changed enough to be within the range included. Therefore, it is nonsensical to try to obtain a conclusion about competitive mechanisms, taking into account the variability in permeabilities and selectivity data, from comparing ideal and real results obtained by different authors under different membrane synthesis procedures and permeation conditions. For instance, Diestel et al. (2015) and Sánchez-Laínez et al. (2015) reported real H_2_/CO_2_ selectivity within the range of 3.0–3.5, but little is known about the membrane performance under pure gas tests [43,46]. Therefore, ideal and real results will be only discussed for those research documents that include both performance analyses.

In ternary H_2_/CO_2_/N_2_ mixtures, changes in N_2_ and H_2_ concentrations maintaining a constant CO_2_ concentration did not affect hydrogen permeability, which depends only on carbon dioxide concentration [47]. This behavior could be extended to quaternary H_2_/CO_2_/N_2_/CO mixtures, where CO did not affect the hydrogen permeability value either. Only changes in CO_2_ concentration displayed a significant effect on hydrogen permeability. This phenomenon was further confirmed when commercial H_2_-selective membranes, such as ULTEM^®^ 100B, Ultrason^®^ and Celezole^®^, were tested under real process compositions [69].

Permeabilities and selectivities towards hydrogen can be improved by manipulating membrane preparation process and permeation conditions. The annealing temperature during the drying process has appeared as one of the factors that can enhance Matrimid^®^ membrane performance. In this sense, Song et al. (2012) identified an increase in hydrogen permeability (32.7 Barrer) up to 230 °C, which matched the maximum H_2_/CO_2_ selectivity (4.1) and almost maximum H_2_/N_2_ and H_2_/CH_4_ selectivities (90.9 and 142.7, respectively) [42]. The annealing procedure influences the mechanical behavior of the membrane, causing an increase in yield and tensile strength; the Young’s modulus and hardness in the pristine membrane; and a decrease in ductility and fracture energy, which favor plastic deformation against fast fracture [57]. A diminishment in ductility causes a diminishment in stretchability and bendability, weakening damage tolerance and mechanical toughness. The presence of residual solvent affects the overall permeability, and even by applying drying temperatures close to boiling point, dynamic mechanical analysis has demonstrated its presence.

When analyzing the experimental conditions, a significant influence of solvent selection and single gas pressure gradient was not found over the permeation properties. In the range of 25–35 °C, temperature influence cannot be distinguished, but it can when it is increased up to 200 °C (Table 2) [58]. H_2_ and CO_2_ permeabilities increase as temperature increases as a consequence of the Arrhenius-type behavior confirmed by experimental permeability values, especially for H_2_; therefore, the higher the temperature is, the better the separation performance [70]. In this sense, the lower CO_2_ permeability increase is connected to a shift from a diffusion-limited regime to a sorption-limited one [19,47]. In spite of the benefit of the high-temperature separation process, it is limited by the Matrimid^®^ glass transition temperature (300–315 °C) and the temperature of industrial waste gas streams [25,69]. For instance, the ammonia industry, steel-making process and methanol production are characterized by low temperatures of 15–45 °C. Zhao et al. (2008) focused, precisely, their research on the temperature effect on gas transport properties [65]. With increasing temperature, gas permeability (H_2_, CO_2_, N_2_ and CH_4_) and H_2_/CO_2_ slightly increased, whereas H_2_/N_2_ and H_2_/CH_4_ decreased. The Arrhenius dependence of permeability allowed the determination of activation energies, following the sequence CO_2_ < H_2_ < CH_4_ < N_2_. Comparable activation energies were obtained for the quaternary mixture H_2_/CO_2_/N_2_/CO following the sequence CO_2_ < H_2_ < CO < N_2_ and, from the results obtained in both studies, the sequence CH_4_ < CO was obtained [47].

Feed pressure had an impact on carbon dioxide permeability, and this was attributed to the solubility dependency of CO_2_, while H_2_, N_2_ and CO are more diffusion dependent [47]. CO_2_ permeability decreased with increasing feed pressure until reaching a minimum value, followed by an increase. This minimum corresponds to the plasticization pressure, common in membranes exposed to highly condensable species, such as CO_2_, H_2_S, H_2_O and hydrocarbons (C_3+_), that causes swelling and disruption of the polymer chain packing and, thus, an increase in FFV [39,71]. When this phenomenon appears, competition between gas solubility and saturation of the polymer sorption sites occurs. The subsequent growth in permeability is associated with the disruption of the polymeric chain packing. On the other hand, sweep gas flowrate affected hydrogen permeability as well, due to the concentration polarization phenomenon in the permeate side; therefore, the minimum flowrate must be maintained [47]. The use of a sweep gas decreases the partial pressure of permeate gases, increasing the driving force and, therefore, the permeability [19].

#### 2.3.2. Hollow Fiber Membranes

Commercial membrane units for gas separation employ both hollow fibers and flat sheet membranes (usually as spiral-wound modules). In addition, the tubular configuration can be used, mainly in the case of membranes supported on a tubular ceramic substrate [20]. The choice of a given membrane geometry depends on the nature of the polymer, the membrane’s performance features and structural strength, the reproducibility, the nature of the separation, the extent of use and the separation economics [28].

Hollow fiber units display some advantages over other membrane geometries, such as their compactness with very high surface areas occupying small volumes; due to the membrane’s high packing density, no membrane support is required, they can be operated at very high pressures and are easier to fabricate [19,28]. Some important weaknesses of HFMs are the pressure drop inside the fibers associated with gas flow through the membrane bore and the concentration polarization in shell-side feed modules, although concentration polarization is well controlled in bore-side feed modules.

The amount of studies analyzing the performance of pure Matrimid^®^ HFMs for gas separation and, particularly, for hydrogen recovery is scarce; therefore, throughout this section, the main observations, results and advances will be presented and discussed.

Studies in the literature have been reported where the cross-sectional analysis of Matrimid^®^ HF fibers displayed a dense (defect-free) skin layer supported on a spongy porous substructure, with macrovoids orientated from the outside to the inside of the HF [55,72].

Peer et al. (2007) found a positive relationship in pure gas permeation experiments when feed pressure was augmented due to the higher driving force [73]. H_2_/CO binary permeation tests displayed the plasticization effects of CO, competitive sorption and concentration polarization effects that appeared when feed pressure was increased. These phenomena led to an increase in carbon monoxide but a decrease in hydrogen permeance. Feed flowrate also had a positive influence on hydrogen permeance. On the contrary, Favvas et al. (2007) considered that their membranes were rigid enough not to be affected by pressure compaction [67], and David et al. (2012) obtained constant permeances and selectivities in the range between 2.3 and 8.0 bar [68].

**Figure 5:**
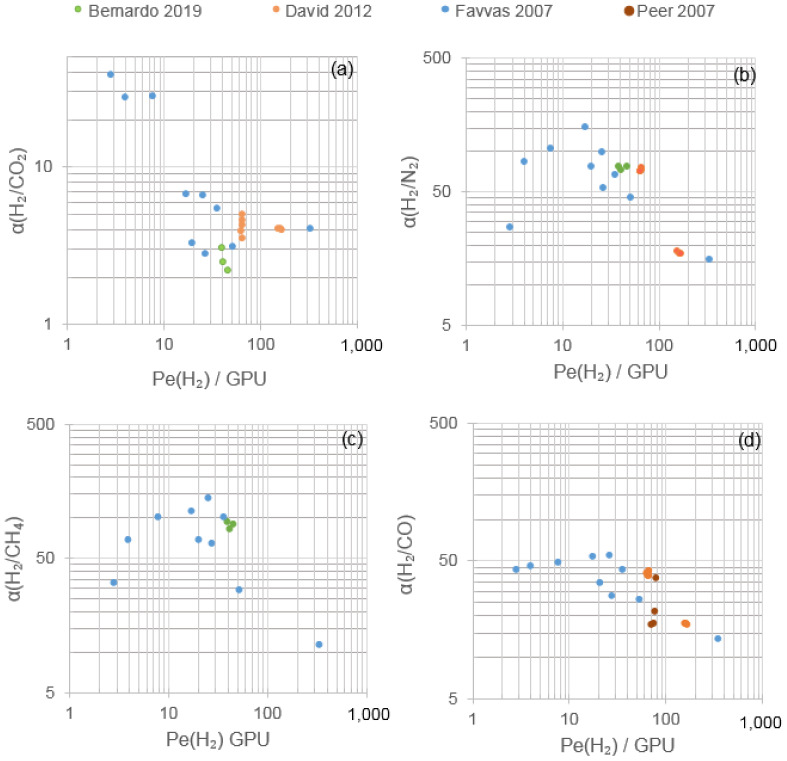
Hydrogen permeance values (GPU) and single gas selectivities obtained for pure Matrimid^®^ hollow fiber membrane (HFMs): (**a**) H_2_/CO_2_, (**b**) H_2_/N_2_, (**c**) H_2_/CH_4_ and (**d**) H_2_/CO. Reference correspondence is found in Table 3.

As occurred in flat sheet Matrimid^®^ membranes, the increase in temperature resulted in an increase in the diffusion of small molecules, i.e., hydrogen, and, consequently, in the selectivity [73]. For instance, working at a feed pressure of 9 bar with a mixture of H_2_/CO 75%/25%, the selectivity increased from 17 (20 °C) to 27 (80 °C) (Figure 5 and Table 3). Modifying the air gap during the membrane fabrication, David et al. (2012) described an increase in permeance values, keeping H_2_/CO_2_ constant but reducing the selectivities of H_2_/N_2_ and H_2_/CO, whose behavior could be attributed to the inadequate contact time for the nascent HF and air/coagulation bath [68].

Favvas et al. (2007) reported a study on the preparation and characterization of Matrimid HF (precursor) and the membranes resulting from a carbonization process. These authors obtained for the polymeric Matrimid^®^ HF selectivities of 14.8, 4.0, 11.2 and 13.2 for H_2_/N_2_, H_2_/CO_2_, H_2_/CH_4_ and H_2_/CO, respectively [72]. H_2_/CO_2_ selectivities were in line with those obtained for flat sheet Matrimid^®^ membranes, but the rest of the pair comparisons were lower, which could be justified by a small fraction of pinholes in the skin layer. The initial spongy porous substructure containing macrovoids was maintained after applying a carbonization process by means of pyrolysis at 650 °C, albeit the number of macrovoids was reduced, and its size was smaller. During the shrinkage of the fibers, the macrovoids reached the outer surface, conferring a higher surface porosity, whereas the inner surface became free of macrovoids. As a consequence, a diminishment in permeance values but an improvement in the separation performance was observed (Table 3), in which the highest H_2_/CO_2_ selectivity (37.8) corresponded to the permeation test developed at 40 °C in the so-called M1 procedure (pyrolysis under N_2_ atmosphere), although hydrogen selectivity against the other gases was poor compared to those detected in flat sheet membranes. Nonetheless, the same membrane at 60 and 100 °C permeation experiments displayed H_2_/CO_2_ selectivities above 27 with ideal selectivities for the rest of the gases comparable with the performance using flat sheet dense membranes (Table 2 and Table 3). From the permeation data, the predominance of the molecular sieving effect was concluded, further confirmed by porosimetry analysis, which is less evident using M2 (pyrolysis under H_2_O atmosphere) and M3 (pyrolysis under CO_2_ atmosphere), characterized by a higher porosity and a lower dense structure and, as a consequence, by higher permeance values.

Another important post-treatment consists of solvent exchange to study the permselective properties and change in the morphology, as reported by Bernardo et al. (2019) [74]. The base results, corresponding to protocol 1 in which there is no solvent exchange, are gathered in Table 3 and display a worse performance in terms of hydrogen recovery than the flat sheet pristine Matrimid^®^. The solvent exchange with methanol and *n*-hexane resulted in the highest permeation increase but a loss in selectivity, whereas a different trend was observed during the exchange with only alcohols. This behavior was attributed to swelling caused by *n*-hexane and the structural damage caused by the quick evaporation of *n*-hexane. Lower H_2_/CO_2_ and H_2_/N_2_ selectivities were observed as the number of carbons in the alcoholic chain and, therefore, the size increase leading to a reduction in the packing density of the membrane.

In a study reported by Peer et al. (2007), the comparison between simulation results by mathematical modelling and experimental data results for binary and multicomponent gas mixtures was useful for model validation under steady-state operating conditions, assuming the plug flow of the gas on the shell and bore side, the same inner and outer diameters and no axial mixing on the bore side [73]. No significant differences between theoretical and experimental results were detected, even assuming constant permeance of the gases. The major differences occurred working at high feed pressures due to the fact that plasticization and concentration polarization effects were not considered in the theoretical model.

### 2.4. Effect of ZIF Addition on Matrimid^®^ MMM’s Performance

Although ceramic and zeolite membranes have displayed exceptional selectivities for many applications, they are not easy to obtain and expensive, so MMMs have arisen as an attractive alternative combining polymer matrix and zeolitic particles, justifying the large number of publications for gas separation by employing these materials [19]. ZIFs, constructed by the tetrahedral coordination of transition metal cations, mainly zinc or cobalt, with imidazole-based ligands, comprise a family of more than a hundred compounds. High specific area, porous channels, availability of structures and easy functionalization are some of the advantages of these materials, providing high selectivity when correctly selected for the proper gas [75]. Among them, ZIF-8, ZIF-90, ZIF-11, ZIF-7 and ZIF-71 constitute the group of ZIFs mostly employed. Throughout this section, the influence of ZIF properties and the performance of ZIF/Matrimid for hydrogen recovery will be analyzed.

#### 2.4.1. Flat Sheet Membranes

**Figure 6:**
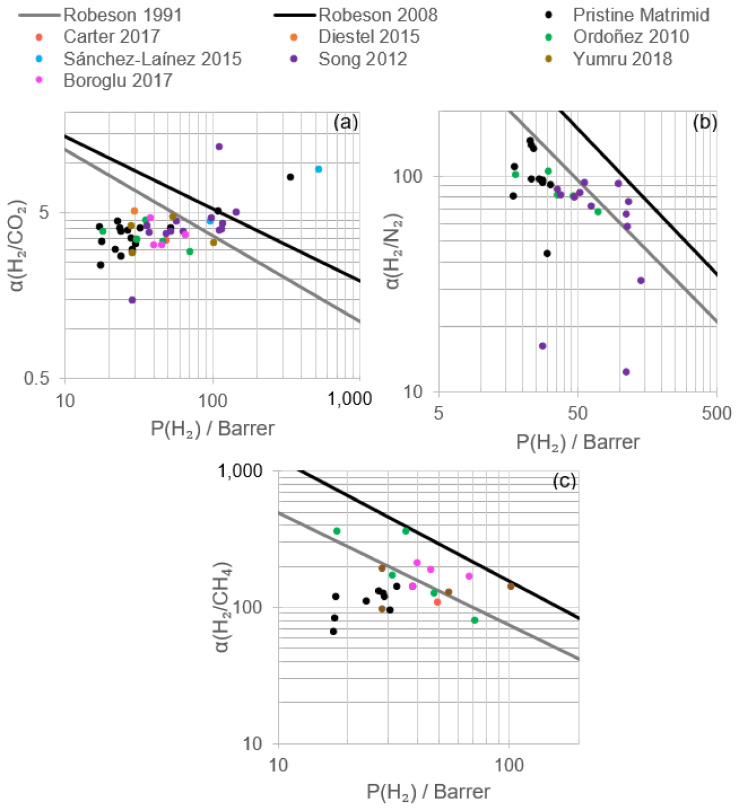
Robeson plot for (**a**) H_2_/CO_2_, (**b**) H_2_/N_2_ and (**c**) H_2_/CH_4_ single gas permeation tests through Matrimid^®^/ZIF mixed matrix membranes (MMMs). Pristine Matrimid refers to the total of the results included in Figure 4. Reference correspondence is found in Table 4.

To date and to the best of our knowledge, only ZIF-8, ZIF-90, ZIF-11 and ZIF-12 have been directly studied for hydrogen recovery blended with Matrimid^®^. It was demonstrated that for the same experimental conditions (i.e., temperature and pressure), Matrimid^®^/ZIF MMMs not only yielded higher permeabilities for hydrogen, carbon dioxide, nitrogen and carbon monoxide compared to pristine Matrimid^®^ membranes but also resulted in higher H_2_/CO_2_ and H_2_/CH_4_ selectivities, whereas H_2_/N_2_ remained almost constant or slightly increased/decreased depending on membrane synthesis conditions (Figure 6 and Table 4). The highest H_2_/N_2_ selectivities were found in the studies by David et al. (2011); however, only pristine Matrimid^®^ was evaluated in this research. Therefore, the likely influence of ZIF on their membranes is unknown [47].

According to the research data shown in Table 4, the Robeson upper bound (2008) for H_2_/CO_2_ was surpassed at high annealing temperatures, as presented by Song et al. (2012), highlighting the selectivity obtained with a 30 wt.% ZIF-8 load and an annealing temperature of 150 °C. High temperature tests at 200 °C and 15 wt.% ZIF-11 resulted in a permeability of 535 Barrer with an optimal H_2_/CO_2_ selectivity of 9.1. The best H_2_/N_2_ performance of a Matrimid^®^ MMM was obtained employing 30 wt.% ZIF-8 and an annealing temperature of 260 °C, almost achieving the Robeson upper bound (2008), albeit diminishing the selectivity compared to pristine Matrimid^®^. With regard to H_2_/CH_4_ separation, ZIF-8, ZIF-11 and ZIF-12 displayed good behavior when mixing with Matrimid^®^, falling over the Robeson upper bound plot (2008), leading ZIF-11 to the highest H_2_ permeability and H_2_/CO_2_ selectivity under comparable membrane preparation and operating conditions. Both ZIF-8 and ZIF-11 do not display structural changes during gas adsorption. According to molecular dynamic simulations, the preferably H_2_ adsorption sites (highest energy) for ZIF-8 are on the top of the imidazolate ring (8.6 kJ/mol) over the C=C bond, and the second adsorption site is located at the center of the channel of the Zn hexagon (6.2 kJ/mol). For ZIF-11, four preferred sites were described: one is located on the benzimidazolate part (13.07 kJ/mol); one on top of the benzene ring (9.86 kJ/mol); and two at the center of the channel of the Zn pentagon (13.03 kJ/mol) and Zn hexagon (5.93 kJ/mol), respectively [76]. The higher adsorption energies found for ZIF-11 could explain the higher permeabilities displayed in the experiments.

**Table 4:** Main bibliographical data in terms of pure gas H_2_, N_2_, CO_2_ and CH_4_ permeabilities and selectivities through Matrimid^®^/ZIF MMMs.

Ref.	*T* (°C)	Δ*P* (Bar)	*P*_H_2__ (Barrer)	*P*_N_2__ (Barrer)	*P*_CO_2__ (Barrer)	*P*_CH_4__ (Barrer)	α_H_2_/N_2__	α_H_2_/CO_2__	α_H_2_/CH_4__	ZIF Load (wt.%/v.%)	ZIF	Comments
Boroglu (2017) [77]	35	4.0	38.3		8.3	0.27		4.6	141.9	10	ZIF-12	
			67.2		18.6	0.40		3.6	168.0	20		
			46.2		14.6	0.25		3.2	184.8	30		
			40.2		12.7	0.19		3.2	211.6	40		
Carter (2017) [48]	35	3.0	48.7	0.61	14.3	0.45	79.8	3.4	108.2	10	ZIF-8	
Diestel (2015) [46]	25	0.2	31.0		9.0			3.4		25	ZIF-8	
			30.0		6.0			5.0		25	ZIF-90	
Ordoñez (2010) [43]	35	1.7	31.2	0.30	9.0	0.18	104.0	3.5	173.3	20	ZIF-8	
			47.2	0.59	14.2	0.38	80.0	3.3	124.2	30		
			71.2	1.05	24.6	0.89	67.8	2.9	80.0	40		
			18.1	0.18	4.7	0.05	100.6	3.8	362.0	50		
			35.8	0.44	8.1	0.10	81.4	4.4	358.0	60		
Sánchez-Laínez (2015) [58]	35	2.0	95.9		21.8			4.4		25	ZIF-11	
	200		535.0		58.8			9.1		15		
Song (2012) [42]	22	4.0	38.1	0.47	10.1	0.26	81.0	3.8	146.3	5	ZIF-8	Annealing 230 °C
			52.6	0.63	13.7	0.45	83.4	3.8	116.8	10		
			63.5	0.88	16.6	0.46	72.2	3.8	138.1	20		
			112.1	1.68	28.7	1.16	66.7	3.9	96.6	30		
			28.9	1.77	19.8	1.06	16.3	1.5	27.3	20		Annealing 60 °C
			36.4	0.42	8.8	0.23	86.6	4.1	158.2	20		Annealing 150 °C
			48.2	0.61	13.0	0.31	79.1	3.7	155.6	20		Annealing 180 °C
			56.5	0.61	12.9	0.36	92.7	4.4	157.0	20		Annealing 200 °C
			113.3	9.21	9.1	8.70	12.3	12.5	13.0	30		Annealing 150 °C
			115.8	2.00	29.2	1.17	57.9	4.0	99.0	30		Annealing 180 °C
			117.3	1.54	27.5	0.97	76.2	4.3	121.0	30		Annealing 200 °C
			98.9	1.08	21.4	0.73	91.6	4.6	135.5	30		Annealing 260 °C
			144.5	4.43	29.2	4.60	32.6	5.0	31.4	30		Annealing 300 °C
Yumru (2018) [62]	35	4	28.1		6.8	0.29		4.2	96.9	10	ZIF-11	
			54.9		11.8	0.43		4.7	127.8	20		
			102.8		31.4	0.73		3.3	140.8	30		
			28.4		10.0	0.15		2.8	189.2	40		

During the microstructural analysis of the Matrimid^®^/ZIF MMMs studied for hydrogen recovery, good dispersion and homogeneity of ZIF particles within the Matrimid^®^ matrix up to 20 wt.% of the filler have been observed [43,57]. From ductile fracture and nanoindentation results, it has been inferred that ZIF particles cause polymeric chain packing modifications and increase Young’s modulus and hardness. Both parameters could serve as quality indicators of particle dispersion homogeneity. Loadings of 80 wt.% directly resulted in the rupture of the MMMs in the permeation cell when applying an upstream pressure [43].

Mahdi and Tan (2016) found in their studies that loadings of ZIF-8 of 10 wt.% provided a better overall dispersion and blending, thus avoiding particle agglomeration and aggregation that is especially relevant over 30 wt.%, although in Matrimid^®^/ZIF-12 (40 wt.%), good dispersion and wetting of ZIF-12 without apparent phase separation and voids were obtained [57,77]. Good dispersion of the fillers was confirmed by SEM analysis, in which a good encapsulation of the particles by a thin layer of Matrimid^®^ was observed. Nanosized ZIF would allow higher loadings owing to the larger surface area, which improves the Matrimid^®^/ZIF interfacial area, although its size was not able to avoid the formation of agglomerated nanocrystals dispersed in the polymer [43]. The addition of fillers resulted in the formation of voids that were not identified in pristine Matrimid^®^ SEM analysis and could be explained by the repulsion between Matrimid^®^ and the fillers [48]. High loads of ZIF would cause an excessive number of nonselective voids that decrease the selectivity towards H_2_, which, to an extent, could be described by a convective-type flow.

A general trend was observed according to the published results: gas permeability increased until reaching a maximum at 20–30 wt.% ZIF load, followed by a decrease in permeability (Table 4). The availability of a higher free volume between polymer chains and, therefore, *d*-spacing would explain the initial trend, whereas a high amount of filler results in the lower availability of polymers for gas transport, diminishing free volume and forcing the gas transport around ZIF nanoparticles [43,62]. The increase in ZIF-8 load resulted in a hydrogen effective diffusion coefficient and solubility increase, which was attributed to the high diffusion and adsorption capacity of this molecule in ZIF-8, whose structure oscillates between “open window” and “close window” [42,78]. Regarding carbon dioxide, only the diffusion coefficient was improved, explaining in this sense the slight increase in H_2_/CO_2_ selectivity. In Matrimid^®^/ZIF-11 membranes, hydrogen and carbon dioxide diffusivity coefficient and solubility increases were, by far, higher than those corresponding to methane: a major increase in diffusion coefficient corresponded to hydrogen, whereas carbon dioxide solubility experienced the highest increase [62].

**Figure 7:**
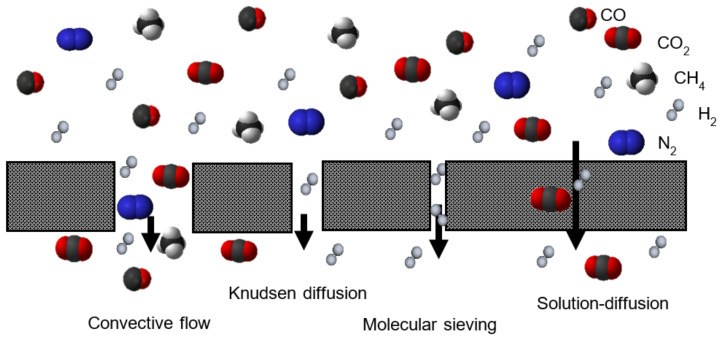
Mechanisms for gas permeation through porous and dense membranes (adapted from Baker (2004) [19]).

A molecular sieving effect (Figure 7) was described by Ordoñez et al. (2010) at ZIF loads above 50%, in which the polymer chain packing could be altered favoring the transport of small molecules such as hydrogen or carbon dioxide and reducing the transport of methane and nitrogen, resulting in higher H_2_/CH_4_ and CO_2_/CH_4_ selectivities [43]. Gas sorption measurements explained the selectivity displayed towards the hydrogen and carbon dioxide of ZIF-based membranes, related to the microporosity of nanosized ZIF whose narrow pore aperture is smaller than the nitrogen or methane molecule kinetic diameter [58]. The slight increase in nitrogen and methane permeability could be explained by the flexible pore structures of ZIF and the increased free volume of the polymer.

When comparing ZIF-8 and ZIF-90 performance, H_2_/CO_2_ selectivity differences could not be explained by diffusion selectivities, because both ZIFs presented a similar topology and pore size (3.4 and 3.5 Å, respectively) [46]. Nonetheless, according to density functional theory studies, the strong interaction between CO_2_ and the aldehyde group of ZIF-90 justified higher H_2_/CO_2_ selectivity. ZIF-8, according to experimental and theoretical studies, was related to an increase in hydrogen permeability, while ZIF-90 was associated with a slight improvement of H_2_/CO_2_ selectivity with a higher permeability. The good performance of Matrimid^®^/ZIF-11 and ZIF-12 can be correlated to ZIF-11 and ZIF-12 pore sizes (3.1 and 3.2 Å, respectively), smaller than those corresponding to ZIF-8 and ZIF-90, which facilitates the selectivity towards hydrogen and carbon dioxide [62,77].

Both annealed and unannealed membranes displayed agglomeration and aggregation when increasing ZIF loadings, the main difference being the absence and presence of trapped solvent, respectively; this is especially important when ZIFs are used as synthetized [42,57]. While Matrimid^®^ and Matrimid^®^/ZIF membranes are yellowish, annealing over 200 °C Matrimid^®^/ZIF-8 resulted in a dark yellowish color, as occurred with ZIF-8, which is likely related to ZIF-8 decomposition [42]. The annealing procedure of MMMs with higher ZIF loadings resulted in the obtention of more brittle membranes, but solvent evacuation permitted the establishment of hydrogen bonds between Matrimid^®^ and ZIF. The cage size and pore volume of ZIFs enable solvents to penetrate inside; therefore, high temperatures, above the boiling point, must be applied in order to eliminate solvent, causing the bond rotation of polyimides, as supported by DMA and TGA measurements. The importance of residual solvents affects the overall permeability, which was confirmed by permeability results when various annealing temperatures were applied: hydrogen permeability at 22 °C increased from 28.9 to 63.5 Barrer when the annealing temperatures increased from 60 to 230 °C (Table 4), chloroform being used as solvent and Matrimid^®^/ZIF-8 MMM composed by a 20 wt.% ZIF-8 [42]. The same trend was observed when the ZIF-8 load was increased to 30%. Nevertheless, nitrogen, carbon dioxide or methane, with a greater molecular size, showed an irregular trend that could be explained by their slower diffusional mass transfer.

Sánchez-Laínez et al. (2015) registered a positive influence of the experimental temperature on H_2_/CO_2_ selectivity, surpassing the Robeson upper bound (2008) at permeation temperatures higher than 100 °C for Matrimid^®^/ZIF-11 membranes and 150 °C for pristine Matrimid^®^ membranes, achieving hydrogen permeabilities of 535 Barrer (Figure 6) [58].

Experiments with gas mixtures are lacking, so there is not extensive knowledge about how gases influence one another and how each gas interact with MMMs based on Matrimid^®^/ZIF. Ordoñez et al. (2010) worked with H_2_/CO_2_ and CO_2_/CH_4_ mixtures (50:50 and 90:10 mol.%, respectively), but the standard deviation between the membranes tested did not allow them to obtain any conclusion about the influence (or not) of working with pure gases or mixtures [43]. On the other hand, Sánchez-Laínez et al. (2015) worked with a mixture of 50:50 mol.% H_2_/CO_2_, but no tests with pure gases were reported [58].

As a consequence of the available results analysis, further research must be conducted in order to optimize the synthesis variables, the experimental conditions and the likely gas mixture–polymer interaction. From these results, a positive influence of filler addition, up to 20 wt.%, on annealing temperature and testing temperature was registered, and their proper optimization displays potential for hydrogen recovery from exhaust gas mixtures.

#### 2.4.2. Mixed Matrix Hollow Fiber Membranes (MMHFMs)

Performance studies on Matrimid^®^/ZIF MMHFMs in hydrogen recovery are lacking; only a few manuscripts that analyze them in propylene/propane separation are available. In these papers, Matrimid^®^ HFMs were prepared by the dry-wet jet spinning process, and ZIFs were added by flowing a solution on the bore side, followed by defect sealing using poly-dimethylsiloxane (PDMS) [79,80].

### 2.5. Towards the Improvement of Matrimid^®^/ZIF Hydrogen Recovery Performance by Polymeric Substitution, Polymeric Blending, Chemical Modification and Filler Substitution or Functionalization

#### 2.5.1. Flat Sheet Membranes

An extensive comparison of the performance of polymeric membranes based on different materials and the use of a wide variety of fillers can be found elsewhere; therefore, this review will focus mainly on alternative materials, methods and procedures that have contributed to the improvement of the hydrogen recovery results already discussed in the previous section [25,33,35,40,75,81,82]. Matrimid^®^ MMMs based on MOFs, such as Cu-414′-BPY-HFS, MIL-53, NH_2_-MIL-53(Al), NH_2_-MIL-101(Al), TKL-107 and FeBTC, led to a maximum hydrogen permeability of 20.3 Barrer and H_2_/CO_2_ selectivity of 2.6, which are comparable to the results already obtained for pristine Matrimid^®^.

**Figure 8:**
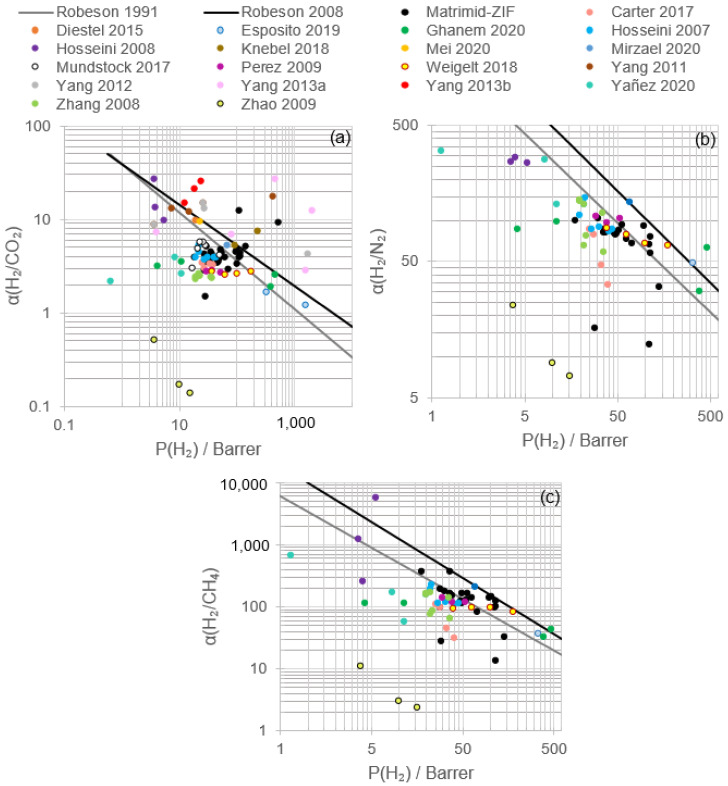
Robeson plot for (**a**) H_2_/CO_2_, (**b**) H_2_/N_2_ and (**c**) H_2_/CH_4_ gas permeation tests through modified Matrimid^®^/ZIF MMMs. Matrimid-ZIF refers to the results included in Figure 6 after ZIF addition to pristine Matrimid. Reference correspondence is found in Table 5.

According to the results included in Figure 8 and Table 5 corresponding to the modification of Matrimid/ZIF membranes, it is possible to achieve better performance and yield from Matrimid^®^ membranes. The combination of ZIF, annealing procedures and blending with other polymers or the selection of optimal operating conditions could guide the process towards exceptional permeation properties. In general terms, the increase in H_2_/CO_2_ and H_2_/N_2_ selectivities occurred, maintaining or lowering hydrogen permeability, whereas higher hydrogen permeability took place at a lower H_2_/CO_2_ selectivity than that showed in Figure 6.

Knebel et al. (2018) reported that the combination of ZIF-8 and ZIF-67 in Matrimid^®^ MMMs caused a hydrogen recovery that was in the Robeson upper bound (2008) (Figure 8) with a hydrogen/carbon dioxide real selectivity of 5.3 and a H_2_ permeability of 94 Barrer at room temperature [83]. Meanwhile, when the operating temperature was elevated till 150 °C, H_2_/CO_2_ selectivity was 7.2 with a hydrogen permeability of 237 Barrer, achieving an attractive zone above the Robeson upper bound (2008) with a compromise between selectivity and permeability. When layers of ZIF-67 and ZIF-67 on the ZIF-8 layer, and ZIF-8 on the ZIF-67 layer, were prepared supported by alumina, H_2_/CO_2_ selectivity was up to 13.2, but H_2_/N_2_ and H_2_/CH_4_ selectivities were in fact low compared to those obtained with pristine Matrimid^®^. It is likely that the sorption of carbon dioxide is more relevant than diffusion selectivity and causes similar values of selectivity independently of the gas.

**Table 5:** Main bibliographical data in terms of pure gas H_2_, N_2_, CO_2_ and CH_4_ permeabilities and selectivities through modified Matrimid^®^/ZIF MMMs in which polymer has been substituted or blended, the MMM has been chemically modified and/or the filler has been substituted or functionalized.

Ref.	*T* (°C)	Δ*P* (Bar)	*P*_H_2__ (Barrer)	*P*_N_2__ (Barrer)	*P*_CO_2__ (Barrer)	*P*_CH_4__ (Barrer)	α_H_2_/N_2__	α_H_2_/CO_2__	α_H_2_/CH_4__	Polymer	Modification
Carter (2017) [48]	35	3.0	30.3	0.70	9.5	0.32	43.3	3.2	94.7	Matrimid^®^	pristine Matrimid
			34.0	0.73	10.5	0.79	46.6	3.2	43.0		Silicalite calcined (10 wt.%)
			28.3	0.36	9.5	0.30	78.6	3.0	94.3		Silicalite uncalcined (10 wt.%)
			40.2	1.19	12.5	1.34	33.8	3.2	30.0		SAPO-34 calcined (10 wt.%)
			25.2	0.29	7.6	0.24	86.9	3.3	105.0		SAPO-34 uncalcined (10 wt.%)
Diestel (2015) [46]	25	0.2	19.0		2.0			9.5		Matrimid^®^	ZIF-90 + ethylendiamine
Esposito (2019) [63]	25		328	6.83	198	9.14	48.0	1.7	35.9	Matrimid^®^/PIM	
			1630	62.8	1380	77.6	26.0	1.2	21.0	PIM	
Ghanem (2020) [84]	35	2.0	4.3	0.05	1.4	0.04	87.1	3.1	108.9	Commercial polyimide from Alfa Aesar	d-PI
			11.2	0.12	3.2	0.10	96.9	3.5	110.2		5 wt.% ZIF-302 d-PI
			386.1	12.9	207.3	12.3	29.9	1.9	31.4		s-PI
			469.2	7.5	186.0	11.1	62.6	2.5	42.3		5 wt.% ZIF-302 s-PI
Hosseini (2007) [56]	35		32.2	0.36	8.3	0.28	89.4	3.9	115.8	Matrimid^®^	20 wt.% MgO untreated
			25.3	0.32	7.4	0.25	79.3	3.4	103.3		20 wt.% MgO, 240 °C (12 h)
			37.6	0.50	10.8	0.39	74.8	3.5	96.7		20 wt.% MgO, 350 °C (1 h)
			41.1	0.52	11.6	0.21	79.0	3.5	199.5		20 wt.% MgO, 350 °C (0.5 h)
			19.8	0.18	5.1	0.13	108.2	3.9	152.3		20 wt.% MgO, silver treatment 2 days
			22.5	0.17	5.1	0.12	130.1	4.5	186.0		20 wt.% MgO, silver treatment 5 days
			22.7	0.16	4.3	0.10	146.5	5.3	222.5		20 wt.% MgO, silver treatment 10 days
Hosseini (2018) [45]	35	H_2_: 3.5 Other gases: 10.0	5.5	0.021	0.6	0.001	260.5	9.4	5500.0	Matrimid^®^/PBI (25/75 wt.%)	
			4.0	0.014	0.3	0.016	288.6	13.1	253.2		*p*-xylene dichloride
			3.6	0.013	0.1	0.003	271.2	26.1	1200.0		*p*-xylene diamine
Knebel (2018) [83]	25	0.5					8.9	6.5	5.5	Ceramic support of α-Al_2_O_3_	ZIF-67
							10.4	12.9	11.4		ZIF-67 on ZIF-8
							9.3	13.2	11.1		ZIF-8 on ZIF-67
			94.0		17.7			5.3		Matrimid^®^	ZIF-8 and ZIF-67
	150		237.0		32.5			7.3			
Mei (2020) [85]	30	4.0	23.3		2.5				9.3	Polysulfone	10 wt.% ZIF-8 with PDA coating
Mirzaei (2020) [44]	25	5.0	68.9	0.51	13.6	0.34	135.9	5.1	201.1	Matrimid^®^	20 wt.% Pd@ZIF-8
Mundstock (2017) [86]	20		17.0		5.7			3.0		Matrimid^®^ supported over Al_2_O_3_	
			50.8		12.9			4.0			NaX
		1.0	21.2		4.5			4.8			PbX
			29.3		5.7			5.2			CuX
			26.0		4.8			5.6			NiX
			23.0		4.2			5.6			Cox
Perez (2009) [87]	35	2.0	29.9	0.28	11.1	0.22	106.8	2.7	135.9	Matrimid^®^	10 wt.% MOF-5
			38.3	0.40	13.8	0.34	95.8	2.8	112.6		20 wt.% MOF-5
			53.8	0.52	20.2	0.45	103.5	2.7	119.6		30 wt.% MOF-5
Sánchez-Laínez (2018) [88]	35	2.0						3.8		Polyamide on P84^®^ support	ZIF-8 (0%*w*/*v*)
	180	2.0						7.9			
	250	2.0						8.4			
	35	2.0						4.4			ZIF-8 (0.2%*w*/*v*)
	180	2.0						9.2			
	250	2.0						11.5			
	35	2.0						9.0			ZIF-8 (0.4%*w*/*v*)
	180	2.0						14.6			
	250	2.0						13.4			
	180	2.0						7.2			ZIF-8 (0.8%*w*/*v*)
Weigelt (2018) [64]	30	1	39.0	0.44	14.5	0.43	88.6	2.7	90.7	Matrimid^®^	8% Activated Carbon
			63.8	0.81	25.6	0.67	78.8	2.5	95.2		31% AC
			101	1.5	39.5	1.06	67.3	2.6	95.3		44% AC
			180	2.8	66.7	2.25	64.3	2.7	80.0		50% AC
Yang (2011) [89]	35	7.1	3.7		0.4			8.7		PBI	pristine PBI
			7.7		0.6			12.9			10 wt.% ZIF-7
			15.4		1.3			11.9			25 wt.% ZIF-7
			26.2		1.8			14.9			50 wt.% ZIF-7 50 wt.% ZIF-7
	180		440.0		25.4			14.6			
Yang (2012) [90]	35	3.5	3.7		0.4			8.6		PBI	pristine PBI
			28.5		2.2			13.0			15 wt.% ZIF-8
			1750		426.6			4.1			60 wt.% ZIF-8
			26.2		1.8			14.6			ZIF-7
Yang (2013) [91]	35	3.5	4.1		0.5			7.1		PBI	pristine PBI
			82.5		6.9			6.8			30 wt.% ZIF-8
			1612.8		397.6			2.8			60 wt.% ZIF-8
	230		470.0		17.9			26.3			30 wt.% ZIF-8
			2015.0		163.8			12.3			60 wt.% ZIF-8
Yang (2013) [92]	35	3.5	12.7		0.9			14.6		PBI	10 wt.% ZIF-90
			18.3		0.9			20.6			25 wt.% ZIF-90
			24.5		1.0			25.0			45 wt.% ZIF-90
Yáñez (2020) [69]	35	5.5	8.4	0.03	2.2	0.05	280.0	3.8	168.0	PEI ULTEM^®^ 1000B	
			11.3	0.09	4.4	0.20	132.4	2.6	56.3	PES ULTRASON^®^ E	
			0.6	0.002	0.3	0.001	322.5	2.2	645.0	PBI Celazole^®^	
Zhang (2008) [49]	25	2.0	17.5	0.22	7.3	0.21	79.6	2.4	83.3	Matrimid^®^	pristine Matrimid
		2.0	19.8	0.14	8.3	0.12	141.3	2.4	164.8		10 wt.% Meso-ZSM-5
		1.5	19.6	0.14	8.5	0.13	139.7	2.3	150.5		10 wt.% Meso-ZSM-5
		2.0	22.2	0.170	8.7	0.130	130.8	2.6	171.0		20 wt.% Meso-ZSM-5
			35.4	0.31	14.6	0.26	114.1	2.4	136.0		30 wt.% Meso-ZSM-5
			36.3	0.62	15.4	0.56	58.6	2.4	64.8		10 wt.% Meso-ZSM-5 (uncalcined)
			22.0	0.34	9.0	0.30	64.8	2.4	73.5		10 wt.% ZSM-5
			23.1	0.30	9.4	0.28	77.1	2.5	82.6		10 wt.% MCM-48
Zhao (2008) [65,93]	35	1.0	3.8	0.16	7.5	0.35	23.7	0.5	10.8	Matrimid^®^	1:0.2 PPG/PEG/PPGDA
			10.0	1.13	59.2	3.36	8.9	0.2	3.0		1:0.5 PPG/PEG/PPGDA
			15.8	2.19	115.8	6.80	7.2	0.1	2.3		1:1 PPG/PEG/PPGDA

The functionalization of ZIF nanoparticles has arisen as a potential solution to improve hydrogen recovery, as pore aperture size can be controlled and/or active catalytic sites can be created, enhancing the gas separation performance [94]. Palladium has gained great attention in hydrogen recovery owing to its high catalytic activity and high hydrogen sorption capacity, which facilitates hydrogen transport through the membrane [44,95,96]. Some advantages of Pd NPs are the reasonable cost; high hydrogen permeability and selectivity; predictable behavior over a long period of time; or resistance to poisoning by H_2_S, Cl_2_, CO, etc. Nevertheless, some disadvantages accompanying Pd NP use in MMMs are associated with their potential aggregation, which can deteriorate the mechanical properties and separation parameters. To avoid Pd NP aggregation, the encapsulation of Pd in ZIF has been proposed. According to Mirzaei et al. (2020), membranes made of Pd@ZIF-8 displayed, in SEM analysis, a relatively uniform dispersion in Matrimid [44]. When adding Pd NPs to the ZIF-8 structure, the permeability of H_2_ (68.9 Barrer, Table 5) was higher than that working with a Matrimid^®^/ZIF-8 MMM (44.7 Barrer), increasing H_2_/CO_2_ ideal selectivity from 3.3 to 5.1, maintaining the permeabilities of CO_2_, CH_4_ and N_2_ constant. This increase was connected to the mechanisms of adsorption, dissociation, association and desorption observed in hydrogen over palladium membranes and the ability of Pd for blocking ZIF-8 pores.

Similar to ZIF functionalization, other fillers, such as zeolite faujasite (FAU), can be functionalized by a wide range of metals. By using alumina supports modified with polydopamine (PDA) and 3-aminopropyltriethoxysilane (APTES), the homogeneous growth in the Matrimid^®^/FAU membrane can be achieved [86]. Usually FAU is synthesized as NaX, but after an ion exchange step with a cobalt, nickel, copper or lead salt, CoX, NiX, CuX or PbX, respectively, are obtained. The addition of Matrimid to form Al_2_O_3_/Matrimid^®^/NaX led to a real H_2_/CO_2_ selectivity of 4.0 (Table 5) higher than that obtained for Al_2_O_3_/Matrimid^®^ (3.0), and this is justified by the interaction of FAU with polar molecules due to its strong electrostatic potential and the accessibility to sodium sites. However, this selectivity (4.0) is lower than that obtained without the addition of Matrimid^®^ (10.3), a phenomenon explained by the weak Matrimid^®^–NaX interaction, the sealing of existing cracks and pinholes and the widening of some of them, as can be inferred from the lower hydrogen and carbon dioxide permeances. Comparing NaX, CoX, NiX, CuX and PbX, the highest selectivity (5.6) corresponded to NIX and CoX, where Ni^2+^ and Co^2+^ had the highest ionic potential, confirming a higher ion–CO_2_ interaction. Although H_2_/CO_2_ selectivity and the corresponding permeabilities are within the results obtained employing Matrimid^®^/ZIF MMMs at room temperature, the performance is still deficient, as the prior upper bound is not reached. Matrimid^®^/AC (activated carbon) MMMs allowed the increase in hydrogen permeability from 31.6 (pristine polymer) to 180 Barrer (50% AC), whereas H_2_/CO_2_ selectivity remained at around 2.7 (below the prior Robeson plot) [64]. Nonetheless, H_2_/N_2_ and H_2_/CH_4_ separation almost reached the Robeson upper bound (Figure 8). Good compatibility of these materials was observed by SEM, where the fillers were covered by a thin layer of polymer without displaying defects. The interaction between Matrimid^®^ and activated carbon could be enhanced by modifying carbon particles.

The usage of a linker in Matrimid^®^/ZIF MMMs has been adopted as a strategy to further improve hydrogen recovery. The covalent binding of ZIF-90 with ethylenediamine and the mixture of this dispersion with Matrimid^®^ solution led to a H_2_/CO_2_ ideal selectivity of 9.5, with a hydrogen and carbon dioxide permeability of 19.0 and 2.0 Barrer, respectively (Table 5) [46]. The addition of the linker meant an improvement of the selectivity compared to Matrimid^®^/ZIF MMM, yet a diminishment in H_2_ and CO_2_ permeabilities (Table 4), surpassing the prior Robeson upper bound (1991). These results could be explained by the elimination in the ZIF-90 crystal surface of the linker distortion of the carboxyaldehyde imidazolate molecules. Considering these advances, it would be interesting to analyze the influence of annealing and permeation temperature. Other linkers, such as poly (propylene glycol) block poly (ethylene glycol) block poly (propylene glycol) diamine (PPG/PEG/PPGDA), suppress the hydrogen transport through the membrane, enriching the permeate stream in carbon dioxide and rendering its composition poor in the retentate (Figure 8) [65,93]. This phenomenon is explained considering that the rubber phase controls the gas permeability instead of the glassy Matrimid^®^.

In recent years, several studies have addressed the options of combining Matrimid with polybenzimidazole (PBI), a polymer that has interesting properties. For this reason, this review describes the characteristics and performance of some membranes based on PBI, and later the studies that report the combination of Matrimid with PBI. Polybenzimidazole (PBI) is a polymeric material selective towards hydrogen that displays good thermal stability [90]. Nevertheless, PBI possesses brittleness and low gas permeance, even in hollow fiber configuration, as a consequence of the rigid polymeric backbone and the high-density chain packing, meaning that it is very difficult to obtain ultrathin PBI membranes [92]. Taking into consideration the literature, Yang et al. (2011) listed six possible modifications that contribute to the enhancement of PBI permeation properties: blending with other polymers, varying the acid moiety during PBI synthesis, crosslinking procedures, thermal rearranging, N-substitution modification and incorporation of inorganic silica NPs [89]. According to the results already published, polymer blending, the incorporation of NPs and crosslinking appear to be feasible options for using PBI in the recovery of hydrogen.

Yang et al. (2011) reported that during the fabrication of PBI/ZIF-7 MMM, good dispersion of ZIF-7 in PBI was obtained up to PBI:ZIF-7 50:50 wt.%, but particle agglomeration was recognized [89]. The addition of ZIF-7 and the increase in the wt.% resulted in an improvement of both ideal and real H_2_/CO_2_ selectivity from 8.7 and 7.1 to 14.9 and 7.2, respectively, with maximum hydrogen permeabilities of 26.2 (single gas) and 13.3 Barrer (mixed gas) at 35 °C (Figure 8 and Table 5). These results are interesting, considering the H_2_ permeability–selectivity relation that surpasses the Robeson upper bound (2008), especially when the operating temperature increased to 180 °C. The increase in hydrogen and carbon dioxide permeabilities was produced by the higher free volume, but, owing to the rigidity of the chains and ZIF-7 accessible cavity diameter (3.0 Å), hydrogen permeability was favored and, consequently, H_2_/CO_2_ selectivity was enhanced. As observed for Matrimid^®^/ZIF, hydrogen and carbon dioxide permeabilities and H_2_/CO_2_ selectivity increased with ZIF-7 content in the polymer matrix.

When PBI was mixed with ZIF-8, H_2_/CO_2_ selectivities up to 14.6 were obtained and a maximum hydrogen permeability of 1750 Barrer was reached at 35 °C with a membrane containing 60 wt.% ZIF-8 [90,91]. Although permeability continuously increased with ZIF-8 content, the maximum selectivity corresponded to 30 wt.%. The combination PBI/ZIF-8 allowed the Robeson upper bound (2008) to be surpassed, but due to the higher accessible cavity of ZIF-8 (3.4 Å), the ideal selectivity was lower compared to PBI/ZIF-7 MMM, albeit higher hydrogen permeabilities could be obtained (Figure 8). Permeation tests at a high temperature (230 °C) resulted in selectivities up to 26.3 (30 wt.%, Table 5) and a maximum hydrogen permeability of 2015 Barrer. A diminishment in mixed gas permeability and selectivity was observed at 35 and 230 °C, but always maintaining the results above the Robeson upper bound (2008), thus exposing the gas transport competition between hydrogen and carbon dioxide [91]. The addition of CO to the mixed gas feed stream did not affect H_2_/CO_2_ selectivity, but hydrogen permeability slightly decreased in 70:30 PBI/ZIF-8. In the 40:60 PBI/ZIF-8 MMM, both permeabilities decreased as a consequence of the competitive sorption and the likely CO pore blocking of the ZIF-8 cavity (CO kinetic diameter 3.76 Å versus cavity diameter 3.4 Å). The assessment of the water effect at different temperatures on the PBI/ZIF-8 MMM displayed a plateau as a result of the thermal stability of the membrane, the water condensation limit at high temperatures and the smaller kinetic diameter of water. The comparison between Matrimid^®^/ZIF-8 (Table 4) and PBI/ZIF-8 (Table 5) usage displays a better performance of the latter in terms of permeabilities and selectivities when they are compared using the same operating temperature and ZIF-8 load.

By using ZIF-90 as a filler, PBI/ZIF-90 exhibited hydrogen permeability and H_2_/CO_2_ ideal selectivity up to 24.5 Barrer and 25.0, respectively, at 55:45 PBI:ZIF-90 and 35 °C (Figure 8 and Table 5) [92]. According to the data, similarly, as explained in Matrimid^®^/ZIF and PBI/ZIF, the higher ZIF-90 content resulted in a better separation performance. ZIF-90 allowed the obtention of higher selectivities at 35 °C compared to ZIF-7 and ZIF-8, which was attributed to the more acidic aldehyde group in ZIF-90 that reinforced the interaction with the alkali N-3 atom on the PBI imidazole ring. Simultaneous transport studies of hydrogen and carbon dioxide showed a diminishment in permeabilities and selectivity compared to ideal gases owing to the competitive sorption, but at 180 °C, hydrogen permeability reached 226.9 Barrer with a selectivity of 13.3.

Taking into consideration the advantages and disadvantages of both Matrimid^®^ and PBI, the performance of the polymeric blending of both materials was assessed by Hosseini et al. (2008) [45]. Miscibility at the molecular level was confirmed by TGA and DSC at various ranges of compositions of the constituents, and it was explained considering the intermolecular interaction by hydrogen bonding between the N–H group of PBI and C=O group of Matrimid^®^. Higher PBI contents resulted in lower permeabilities but higher H_2_/CO_2_, H_2_/N_2_ and H_2_/CH_4_ selectivities, reaching selectivities of 9.4, 260.5 and 5500, respectively (Figure 8 and Table 5), and this was associated with the free volume provided by Matrimid^®^ (0.268, pure) and PBI (0.116, pure). In spite of the similar structure, the lateral oxygen and methyl groups of Matrimid^®^ avoid polymeric chains to be closer. When both polymers are blended, hydrogen bonding limits the mobility of the polymer chains and decreases the interstitial distances. Although the obtained results are promising, and selectivities are higher than those corresponding to pristine Matrimid^®^, Matrimid^®^/ZIF or even some pristine PBI and PBI/ZIF, they are still beneath the Robeson upper bound (2008). The chemical modification of Matrimid^®^ carboxyl and PBI N–H groups was also proposed by Hosseini et al. (2008) to enhance Matrimid^®^/PBI membrane performance [45]. A crosslinking procedure was developed evaluating *p*-xylene dichloride and *p*-xylene diamine that reacts with PBI N–H groups and Matrimid^®^ amide groups, respectively. The modification process resulted in a sharp decrease in the permeabilities of all gases but selectivities of hydrogen improved, especially at prolonged treatment times. Gas transport performance was better by chemical modification employing *p*-xylene dichloride and confirmed by XRD analysis in which the interstitial space shifted from the original value.

Yáñez et al. (2021) reported that working at near-ambient temperatures (35 °C), ULTEM^®^ 1000B polyetherimide (PEI) and Ultrason^®^ E polyethersulfone (PES) exhibited better performance than Celazole^®^ PBI (Table 5) in both pure and mixed gas permeation tests, surpassing the prior Robeson upper bound (1991) in the case of H_2_/N_2_ and H_2_/CH_4_ mixtures, but far from the performance displayed by Matrimid^®^/ZIF and PBI/ZIF. No competitive sorption among hydrogen and nitrogen, carbon monoxide or methane was described apart from when carbon dioxide was present [69]. Other polyimides and modified polysulfones, even incorporating ZIF-302 and ZIF-8, respectively, displayed lower selectivities and permeabilities than those described for pristine Matrimid^®^ and PBI (Figure 8 and Table 5) [84,85,97]. However, by applying the proper preparation conditions, high hydrogen permeabilities could be achieved by the polyimide membrane, reaching selectivities of H_2_/N_2_ and H_2_/CH_4_ that surpass the Robeson upper bound (2008), although H_2_/CO_2_ selectivity was lower than 2.5, compromising the final target of separating hydrogen from carbon dioxide [84]. Polyimide membranes based on 3,3′,4,4′-Benzophenonetetracarboxylic dianhydride monomer displayed attractive selectivities up to 9.8 but lowering permeability down to 1.7 Barrer [98]. The employment of polyamide on P84^®^ polyimide support allowed the obtention of H_2_/CO_2_ selectivities up to 8.4 and hydrogen permeances around 988 GPU, whereas P84^®^/ZIF-8 nanocomposite membranes reached a selectivity of 18.1 at 180 °C, 0.4%(*w/v)* ZIF-8 and 6 bar without transmembrane total pressure differences [88]. The polymer of intrinsic microporosity PIM-EA(H2)-TB enabled the achievement of H_2_ permeability of 1630 Barrer, but H_2_/CO_2_ and H_2_/N_2_ selectivities remained under 1.5 and 26, respectively [63]. Its blending with Matrimid^®^ resulted in 328 Barrer for hydrogen but poor selectivities (1.7 and 48, respectively). Additionally, in both the pure polymer and blend, the prior Robeson line was surpassed, and the upper one was reached for H_2_/CH_4_ separation (Figure 8).

Finally, it is worthwhile to compare how other fillers combined with Matrimid^®^ performed hydrogen recovery. Silicate, SAPO-34, MgO, ZSM-5 and MOF-5 are some of the fillers that improved pristine Matrimid^®^ effectiveness. Silicate and SAPO-34 are microporous materials with pore sizes equal to 5.5 and 3.8 Å, respectively, higher than the corresponding to ZIF-8 (3.4 Å) [48]. Matrimid^®^/silicate and Matrimid^®^/SAPO-34 MMMs with uncalcined or calcined fillers, according to SEM analysis, seemed to be homogenously clustered (silicate) and dispersed (SAPO-34), whereas two different distribution patterns were detected for ZIF-8: clustering and homogeneous dispersion. Gas transport through Matrimid^®^/uncalcined filler MMMs occurred only through interfacial voids between Matrimid and the filler, and the Matrimid^®^ free volume caused by the chain mobility, leading to permeabilities lower than those obtained in pristine Matrimid^®^, but higher than the calcined ones (Table 4 and Table 5). In terms of selectivity, H_2_/CO_2_ selectivity slightly decreased for uncalcined and calcined silicate and SAPO-34; H_2_/N_2_ selectivity slightly increased for calcined fillers but drastically decreased for uncalcined fillers; and H_2_/CH_4_ selectivity diminished gradually by using calcined fillers and sharply when uncalcined fillers were selected. Both materials led to a recovery performance poorer than ZIF-8 that was explained considering the SEM analysis in which a better integration of ZIF-8 in the MMM was observed. The reduction in gas permeability in uncalcined fillers was related to their nonporous nature, diminishing the availability of Matrimid^®^ free volume.

According to Hosseini et al. (2007), the employment as a filler of MgO resulted in an homogeneous dispersion of MgO in Matrimid^®^ and contributed to the enhancement of H_2_/CO_2_ selectivity and gas permeabilities, especially at the highest MgO load (40 wt.%), albeit H_2_/N_2_ and H_2_/CH_4_ selectivities suffered a diminishment [56]. This increase, like other MMMs already described, was associated with the interfacial microvoids between the polymer and the filler. Nevertheless, the large size of MgO pores (40 Å) hindered the selectivity towards specific gas molecules. Two strategies were followed to improve Matrimid^®^/MgO results: heat treatment and silver treatment. By heat treatment, two behaviors were described, i.e., at 240 °C, there was a diminishment in permeabilities and selectivities, whereas at 350 °C, the permeabilities increased (Table 5). This was attributed to the arrangement of the polymer chains, diminishing the free volume below T_g_ and increasing above *T*_g_ due to the higher mobility. A positive influence of the rapid cooling once temperature was applied for enough time was also detected, owing to the instantaneous freezing of free volume. MMMs with MgO treated with silver and annealed at 150 °C displayed worse permeabilities than Matrimid^®^/MgO (20 wt.%) but higher selectivity towards hydrogen, especially enlarging the treatment time. This behavior was explained by the size exclusion mechanism caused by silver ions. Despite the improvements achieved with MgO and its modification, the results were still below the prior Robeson upper bound (2008) and, therefore, the performance was worse than that obtained by using ZIF-based MMMs (Figure 8).

On the other hand, ZSM-5 and MOF-5 constitute examples of fillers that contribute to better hydrogen recovery from gas streams containing nitrogen and methane but did not have any effect on, or even worsened, the hydrogen/carbon dioxide separation (Table 5) [49,87]. Increasing ZSM-5 content resulted in a higher chain rigidification and due to the narrow pore size, the gas diffusion slowed, and thus, permeabilities diminished. No competitive transport was detected when working with gas mixtures (Y. Zhang et al., 2008). However, the opposite phenomena occurred with MOF-5 where higher loads led to higher permeabilities, while selectivities remained constant, which could be explained by the porosity of the MOF-5 [49,87].

From all the information gathered here, the enormous efforts made and the need for a parameter study to further enhance the performance of Matrimid and PBI membranes can be highlighted, but undoubtedly, both have shown magnificent separation properties, providing a new generation of material combinations whose yield is above the Robeson upper bound stablished in 2008.

#### 2.5.2. Hollow Fiber Membranes

Although more information can be found for H_2_/N_2_, O_2_/N_2_, CO_2_/N_2_ and CO_2_/CH_4_ gas separation by polymeric HFMs, the study of H_2_/CO_2_ is still limited, perhaps owing to the relatively poor H_2_/CO_2_ selectivity already explained and caused by diffusion–solubility competition and the possible CO_2_-induced plasticization [99]. Consequently, different strategies have been proposed to improve the membrane performance and will be analyzed in this section. As previously discussed, the combination of Matrimid^®^ with PBI may achieve better gas separation properties, exploiting the advantages of both polymers and overcoming the drawbacks attributed to their use.

During the preparation of the Matrimid^®^/PBI blend HFM, despite the appropriate cross-sectional circularity and concentricity, a nonporous structure was obtained [68,100]. The addition of a nonsolvent, such as methanol, led to a sponge-like structure with a small pore size and an extremely small inner fiber diameter. Furthermore, the mechanical stability was poor, identifying its brittleness when dense films were fabricated using Matrimid^®^/PBI blend.

In this sense, an attractive alternative reported by Hosseini et al. (2010) is the synthesis of dual-layer hollow fibers using polysulfone as an inner-layer that confers a good mechanical support and an asymmetric structure that decreases the gas transport resistance [99]. The outer-layer blend is then composed of a Matrimid^®^/PBI blend, and the HFM is prepared by co-extrusion using the dry-jet wet-quench spinning process depicted in Figure 3. SEM analysis displayed two different morphologies in every HF corresponding to the asymmetric structure in the outer layer formed by spongy-like cells surrounded by a thin-selective layer without macrovoids, and the thicker inner layer presenting open cell pores and finger-like macrovoids [99,101]. The low failure and structural collapse susceptibility was ensured by the absence of delamination between the inner and outer layers, which was justified by the good miscibility at the molecular level between Matrimid^®^ and PBI (formation of hydrogen bonds) and the compatibility of the inner and outer dopes in terms of solvent interdiffusion and similar solubility parameters.

**Figure 9:**
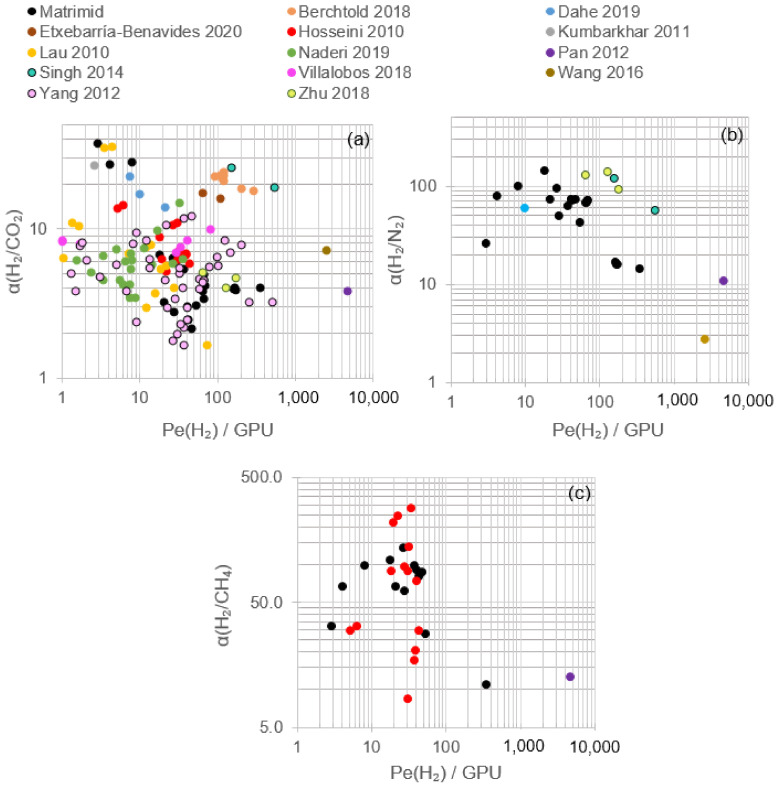
Hydrogen permeance values (GPU) and selectivities obtained for modified Matrimid^®^ HFMs: (**a**) H_2_/CO_2_, (**b**) H_2_/N_2_ and (**c**) H_2_/CH_4_. Reference correspondence is found in Table 6.

In the study reported by Hosseini et al. (2020), higher hydrogen permeances and H_2_/CO_2_ selectivities were obtained compared to pure Matrimid^®^ HFMs (Figure 9, Table 3 and Table 6) [99]. It was found that the highest hydrogen permeance occurred without air gaps (43.2 GPU) in the membrane directly coagulated by water, producing the sudden polymer chain contraction without any possibility of configuration change and resulting in the presence of a larger amount of fine pores, higher free volume and, therefore, lower selectivity. Introducing an air gap and enlarging its length led to an increase in H_2_ and CO_2_ and a diminishment in CH_4_ permeances, and higher H_2_/CO_2_ and H_2_/CH_4_ selectivities, caused by gravity-induced chain orientations that complicate the transport of bigger molecules controlling the gas transport by the diffusion selectivity. Sample IDs C and D in the comments column in Table 6 for the results of Hosseini et al. (2010) display the transport properties effect of allowing the free fall of the nascent fiber or spinning it with elongational draw. Elongational draw resulted in an enhanced gas separation, except for H_2_/CO_2_ in the coated HFM. According to the data presented by Hosseini et al. (2010), elongational drawing had a major influence on the fiber microstructure compared to air gap modification. Higher elongational draw ratios are usually associated with a higher productivity and lower HF diameter, as well as the modification of the separation performance and membrane morphology [102].

The application of a silicon rubber coating allowed the sealing of defects in the dense-selective layer and, as a consequence, the obtention of higher selectivities (Table 6) despite the slight decrease in permeance values, providing better performance than pristine Matrimid^®^ HFMs [99]. The chemical crosslinking modification of the HFM with *p*-xylylene diamine caused a sharp decline in permeance and H_2_/CH_4_ selectivity but positively affected H_2_/CO_2_ selectivity, especially at short treatment times (14.3 versus 6.8 in unmodified HFM) [99]. These results clearly demonstrated the promotion of diffusion selectivity after the chemical modification but mainly penalizing free volumes and interstitial distances between polymer chains. On the other hand, Lau et al. [101] reported that the vapor phase modification of PBI-Matrimid^®^/polysulfone with ethylenediamine converted imide groups into amides, reducing the *d*-space from 5.17 to 5.07 Å, causing a diminishment in permeance values (hydrogen permeance was reduced to the half of the original) and an increase in H_2_/CO_2_ selectivity (almost doubled).

The increase in the outer-layer dope flow rate led to a decrease in permeance values and H_2_/CO_2_ selectivity but an increase in H_2_/CH_4_ and CO_2_/CH_4_ selectivities, which is explained considering the obtention of thicker membranes with larger gas transport resistance.

Long-term aging studies in Matrimid^®^ double skin layer HFMs displayed a diminishment in the gas permeance after 30 months as a consequence of polymer chain relaxation in thin films and the densification of the polymer matrix, which causes lower free volume availability, eliciting a size-sieving effect in molecules such as H_2_/N_2_ that enhances selectivity [103]. This phenomenon was observed either with or without ethylendiamine crosslinking.

**Table 6:** Main bibliographical data in terms of pure gas H_2_, N_2_, CO_2_ and CH_4_ permeance (Pe) and selectivities through modified Matrimid^®^ HFMs in which polymer has been substituted or blended, the HFM has been chemically modified and/or the filler has been substituted or functionalized.

Ref.	*T* (°C)	Δ*P* (Bar)	*P*e_H_2__ (GPU)	*P*e_N_2__ (GPU)	*P*e_CO_2__ (GPU)	*P*e_CH_4__ (GPU)	α_H_2_/N_2__	α_H_2_/CO_2__	α_H_2_/CH_4__	Polymer or Ceramic Material	Comments
Berchtold (2016) [104]	250	1.4	118		4.9			24.0		PBI/polysulfone	Feed pressure influence
		6.9	110		4.8			23.0			
		10.3	120		5.2			23.0			
		13.7	120		5.7			21.0			
	225		92		4.1			22.4			Temperature influence
	250		116		5.3			22.0			
	300		198		10.5			18.8			
	350		285		15.9			17.9			
Dahe (2019) [105]	250	1.4	9.7	0.4	0.6		24.1	17.1		PBI	HFM-1 21.3% PBI (acetone); % outer coagulant 0.5 v.% water (acetone)
			21.0	1.1	1.5		18.4	14.0			HFM-1 20.0% PBI (acetone); % outer coagulant 2.0 v.% water (acetone)
			7.6	0.1	0.3		62.0	22.4			HFM-1 21.5% PBI (acetone/ethanol 15/85); % outer coagulant 2.0 v.% water (acetone)
Etxebarría (2020) [106]	150	7.0	65		3.7			17.6		PBI	no fillers
			107		6.6			16.1			10 wt.% ZIF-8
Hosseini (2010) [99]	35	H_2_: 3.5 other gases: 10	43.2		7.3	1.46		5.9	29.6	Matrimid^®^/PBI	A before silicone rubber coating
			30.3		4.9	3.54		6.2	8.6		C before silicone rubber coating
			36.5		5.5	2.13		6.7	17.2		X before silicone rubber coating
			38.7		5.7	1.85		6.8	20.9		Y before silicone rubber coating
			31.6		4.4	0.22		7.2	141.5		A after silicone rubber coating
			17.8		2.0	0.20		9.0	89.6		C After silicone rubber coating
			26.5		2.5	0.27		10.6	96.9		X After silicone rubber coating
			29.3		2.6	0.33		11.1	89.2		Y After silicone rubber coating
			39.0		5.8	0.53		6.8	74.0		D before silicone rubber coating
			32.7		4.8	0.12		6.8	284.0		D after silicone rubber coating
			22.1		4.2	0.09		5.2	245.2		B before silicone rubber coating
			18.9		3.0	0.09		6.4	222.2		B after silicone rubber coating
			6.1		0.42	0.19		14.5	32.6		Y crosslinking 0.5 s
			5.1		0.37	0.17		13.9	29.7		Y crosslinking 1.0 min
			0.6		0.06	0.04		9.2	16.1		Y crosslinking 5.0 min
Kumbharkar (2011) [107]	100	5–8	0.3		0.046			7.2		PBI	
	200		0.6		0.048			12.9			
	300		1.0		0.046			21.5			
	400		2.6		0.096			27.1			
Lau (2010) [101]	35	1.4	72.6		42.97			1.7		6FDA-NDA/PES dual layer	Original
			12.1		4.05			3.0			Vapor phase modification (VPM) Method A 2 min
			3.4		0.10			34.8			VPM Method A 5 min
			27.7		6.88			4.0		Matrimid^®^/PBI	Original
			18.6		3.42			5.4			VPM Method A 2 min
			11.9		1.56			7.6			VPM Method A 5 min
			7.1		1.03			6.9		Torlon^®^	Original
			1.6		0.16			10.4			VPM Method A 2 min
			0.1		0.03			4.8			VPM Method A 5 min
			15.4		4.13			3.7		6FDA-NDA/PES dual layer	VPM Method B 2 min
			4.4		0.13			35.5			VPM Method B 5 min
			21.7		3.77			5.8		Matrimid^®^/PBI	VPM Method B 2 min
			13.8		1.77			7.8			VPM Method B 5 min
			1.3		0.12			11.0		Torlon^®^	VPM Method B 2 min
			1.0		0.16			6.4			VPM Method B 5 min
Naderi (2019) [108]	25	7.0	2.36		0.46			5.1		Dual layer Inner layer: polysulfone Outer layer: Polyphenylsulfone/PBI	HSP-0: PBI/DMAc/LiCl 22/79.8/1.2 (wt.%). Before silicon rubber coating
			5.50		1.22			4.5			HSP-5: (PBI/sPPSU 95:5)/DMAc/LiCl 22/79.8/1.2 (wt.%). Before silicon rubber coating
			7.52		1.75			4.3			HSP-10: (PBI/sPPSU 90:10)/DMAc/LiCl 22/79.8/1.2 (wt.%). Before silicon rubber coating
			8.78		2.53			3.5			HSP-20: (PBI/sPPSU 80:20)/DMAc/LiCl 22/79.8/1.2 (wt.%). Before silicon rubber coating
			1.54		0.25			6.2			HSP-0 after silicon rubber coating
			3.39		0.74			4.6			HSP-5 after silicon rubber coating
			6.14		1.42			4.3			HSP-10 after silicon rubber coating
			7.44		2.14			3.5			HSP-20 after silicon rubber coating
			7.6		1.4			5.5			HSP-10-40 thermal treatment 40 °C
			7.8		1.3			6.2			HSP-10-80 thermal treatment 80 °C
			7.6		1.1			6.8			HSP-10-120 thermal treatment 120 °C
			5.0		0.7			7.3			HSP-10-120 chemical crosslinking 3% DBX
			3.4		0.5			6.6			HSP-10-120 chemical crosslinking 6% DBX
	30	14.0	13.8		2.4			5.8			Mixed gas. HSP-10-120-30
	60		26.1		4.4			5.9			Mixed gas. HSP-10-120-60
	90		35.6		5.7			6.3			Mixed gas. HSP-10-120-90
	30		6.4		1.1			6.1			Mixed gas. HSP-10-3%DBX-120-30
	60		11.3		1.5			7.4			Mixed gas. HSP-10-3%DBX-120-60
	90		16.7		1.7			9.7			Mixed gas. HSP-10-3%DBX-120-90
	180		32.1		2.2			14.9			Mixed gas. HSP-10-3%DBX-120-180
Pan (2012) [109]	22	1.0	4598	418	1194	358	11.0	3.9	12.8	ytria-stabilized zirconia	ZIF-8
Singh (2014) [110]	250		540.0	9.3	28.4			58.0	19.0	PBI	
			150.0	1.3	5.8			120.0	26.0		
Villalobos (2018) [111]	35		0.05		0.01			4.8		PBI	Pristine
	45		0.07		0.01			5.0			
	60		0.09		0.02			5.3			
	22		29.0		4.14			7.0			0.05 M Pd NPs
	35		34.0		4.47			7.6			
	45		40.0		4.71			8.5			
	60		80.0		8.00			10.0			
	22		0.55		0.06			9.0			0.1 M Pd NPs
	35		1.0		0.12			8.5			
	45		1.0		0.12			8.3			
	60		1.65		0.21			8.0			
Wang (2016) [112]	20	2.5	2493.3	886.8	343.4		2.8	7.3		Silicon nitride ceramic	ZIF-8
Yang (2012) [90]	25	3.5	1.3		0.3			5.0		Dual layer: inner Matrimid^®^; outer PBI/ZIF-8	PZM00-MA 0% ZIF-8. Solvent-exchange: methanol. Single gas
			0.8		0.1			6.2			PZM00-MB 0% ZIF-8. Solvent-exchange: methanol. Single gas
			0.8		0.1			7.0			PZM00-MC 0% ZIF-8. Solvent-exchange: methanol. Single gas
			1.7		0.2			7.7			PZM00-IA: 0% ZIF-8. Solvent-exchange: isopropanol. Single gas
			2.1		0.3			6.2			PZM00-IB: 0% ZIF-8. Solvent-exchange: isopropanol. Single gas
			1.8		0.2			8.2			PZM00-IB: 0% ZIF-8. Solvent-exchange: isopropanol. Single gas
			6.6		1.7			3.9			PZM10-MA 10% ZIF-8. Solvent-exchange: methanol. Single gas
			0.9		0.1			6.6			PZM10-MB 10% ZIF-8. Solvent-exchange: methanol. Single gas
			1.5		0.4			3.8			PZM10-MC 10% ZIF-8. Solvent-exchange: methanol. Single gas
			13.3		2.1			6.3			PZM10-IA: 10% ZIF-8. Solvent-exchange: isopropanol. Single gas
			8.9		0.9			9.5			PZM10-IB: 10% ZIF-8. Solvent-exchange: isopropanol. Single gas
			13.2		2.4			5.5			PZM10-IB: 10% ZIF-8. Solvent-exchange: isopropanol. Single gas
			8.9		3.7			2.4			PZM20-MA 20% ZIF-8. Solvent-exchange: methanol. Single gas
			21.0		4.6			4.6			PZM20-MB 20% ZIF-8. Solvent-exchange: methanol. Single gas
			57.4		12.4			4.6			PZM20-MC 20% ZIF-8. Solvent-exchange: methanol. Single gas
			28.3		8.2			3.5			PZM20-IA: 20% ZIF-8. Solvent-exchange: isopropanol. Single gas
			32.2		6.4			5.0			PZM20-IB: 20% ZIF-8. Solvent-exchange: isopropanol. Single gas
			66.8		14.5			4.6			PZM20-IB: 20% ZIF-8. Solvent-exchange: isopropanol. Single gas
			36.0		21.5			1.7			PZM33-MA 33% ZIF-8. Solvent-exchange: methanol. Single gas
			248.9		77.5			3.2			PZM33-MB 33% ZIF-8. Solvent-exchange: methanol. Single gas
			497.6		152.4			3.3			PZM33-MC 33% ZIF-8. Solvent-exchange: methanol. Single gas
			22.7		7.6			3.0			PZM33-IA: 33% ZIF-8. Solvent-exchange: isopropanol. Single gas
			34.9		8.7			4.0			PZM33-IB: 33% ZIF-8. Solvent-exchange: isopropanol. Single gas
			32.0		5.8			5.5			PZM33-IB: 33% ZIF-8. Solvent-exchange: isopropanol. Single gas
	25	6.0	3.0		0.6			4.8			PZM10-IB, 10% ZIF-8. Mixed gas
	35		5.0		0.9			5.8			
	50		8.0		1.0			8.0			
	80		12.0		1.4			8.5			
	120		22.0		2.1			10.7			
	145		37.0		3.1			11.8			
	180		45.0		3.7			12.2			
	25		26.0		14.4			1.8			PZM20-IB 20% ZIF-8. Mixed gas
	35		30.0		15.0			2.0			
	50		40.0		16.0			2.5			
	80		58.0		14.5			4.0			
	120		76.0		13.6			5.6			
	145		99.0		15.2			6.5			
	180		123.0		14.8			8.3			
	25		36.0		16.4			2.2			PZM33-IB 33% ZIF-8. Mixed gas
	35		34.0		14.8			2.3			
	50		40.0		13.3			3.0			
	80		65.0		14.8			4.4			
	120		100.0		17.5			5.7			
	145		145.0		20.7			7.0			
	180		201.0		25.8			7.8			
Zhu (2018) [113]	35.0	5.0	63.3	0.5	12.2		132.0	5.2			Pure
			172.2	1.8	36.5		94.1	4.7		Ultem^®^ polyetherimide	15% MIL-53
			127.1	0.9	31.4		144.5	4.1			15% S-MIL-53

Although no permeation results were provided, Li et al. (2004) presented an interesting and thorough analysis of co-extruded Matrimid^®^/PES dual-layer HFMs and the main variables affecting the HFM morphology and performance [102]. As explained for Matrimid^®^/PBI/polysulfone, Matrimid^®^/PES did not present delamination at the interface of the dual layer, displaying, in SEM analysis, an outer dense layer and an inner porous surface. The dense macrovoid-free layer formed by Matrimid^®^ remained, even when changing the inner-layer dope composition and spinning conditions, as occurred in the study of David (2012) [100]. This phenomenon has been associated with the high viscosity and poor fluidity of Matrimid^®^ when in contact with nonsolvents, and the structure-transition thickness, defining the critical structure-transition thickness as the value that displays a change from a finger-like (above critical thickness) to a sponge-like structure (below critical thickness). The value of this variable depends on certain parameters, such as dope and membrane formulation and the membrane materials used.

A good tradeoff between hydrogen permeation (2493 GPU) and H_2_/CO_2_ selectivity (7.3) was obtained by a continuous ZIF-8 membrane on the outer surface of silicon nitride HF (Figure 9 and Table 6), resulting in one of the highest permeances with a selectivity higher than those presented for pristine Matrimid^®^ dense and HF membranes, but not for nitrogen (H_2_/N_2_ selectivity equals to 2.8) [112]. These results were attributed to the blocking effect caused by the adsorbed CO_2_ that stablish cooperative interactions with other carbon dioxide molecules, but not with hydrogen or nitrogen.

**Figure 10:**
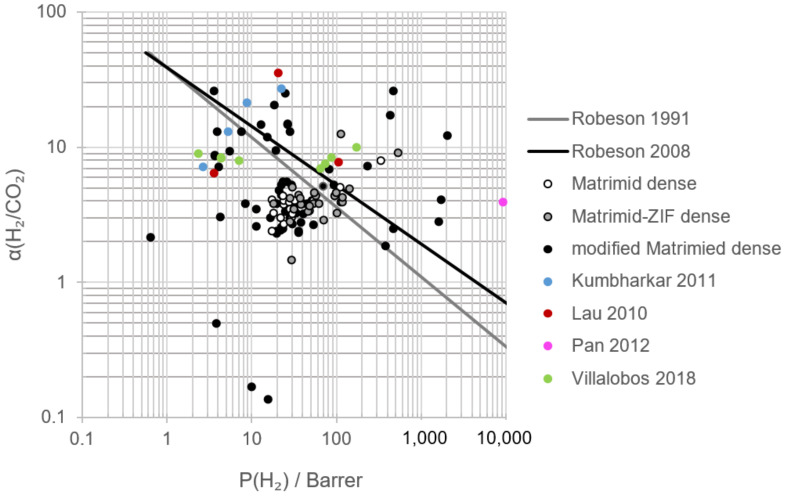
Robeson plot for H_2_/CO_2_ permeation tests: performance comparison between HFMs and flat sheet membranes.

In HFMs fabricated with PBI, a clear influence of bore fluid composition was reported by Kumbharkar et al. (2011) [107]. When bore fluid was composed by a water/*N*,*N*-dimethylacetamide (DMAc) ratio of 10/80 (wt.%), carbon dioxide was not detected in the permeate stream, and very low hydrogen permeance (1.4 GPU, Figure 9 and Table 6) was measured as a consequence of the sublayer resistance caused by the skin layer formation on the inner surface of the HF. It could be overcome by increasing the DMAc ratio up to 50 wt.%, increasing from 0.3 and 7.2 (100 °C) to 2.6 and 27.1 (400 °C) hydrogen permeance (GPU) and H_2_/CO_2_ selectivity, respectively. This selectivity entails one of the highest gathered during the elaboration of this review, and it is a consequence of the rigidity and the defect-free HFM synthesized, but the main challenge is being faced with the low permeance value. Furthermore, considering the thickness measured from SEM analysis, the permeability increased up to 22.9 Barrer (selectivity 27.1), which places this HFM in the separation attractive area, above the Robeson upper bound (2008), improving the performance observed for many flat sheet membranes. For a matter of comparison, Figure 10 includes the permeabilities described in previous figures for flat sheet membranes and those corresponding to HFMs when authors provided approximated thickness from SEM and TEM images.

Consequently, higher hydrogen permeances are required and implied some strategies, such as the minimization of the dense selective layer thickness, minimization of gas phase resistance and development of high porous inner surfaces. In order to obtain high selectivities, the selective layer needs to be defect free, and macrovoids in the support layer need to be minimized. In an interesting study by Singh et al. (2014), the authors prepared a HFM with PBI selective layers between 160 and 2180 nm, after defect sealing, and subsequently carried out permeation tests operating at syngas temperatures (250 °C) close to water–gas shift reactors [110]. Attractive hydrogen permeances higher than 500 GPU and H_2_/CO_2_ and H_2_/N_2_ selectivities equal to 19 and 58, respectively, were obtained for thinner selective layers (Figure 9 and Table 6). Membranes with a thicker selective layer yield a hydrogen permeance value and H_2_/CO_2_ and H_2_/N_2_ selectivities of 150 GPU, 26 and 120, respectively. In a more recent work from the same research group, a nonsolvent chemistry sensibility assessment was developed taking into consideration the solvent/nonsolvent solubility parameters, which include the contribution of dispersion, polar and H-bonding forces, and solvent/nonsolvent diffusion [105]. SEM images of PBI HFMs fabricated using PBI/LiCl/DMAc displayed two different microstructures due to the diffusion features—nonsolvent dope solution: highly porous membranes were obtained using as nonsolvent acetone, ethanol, isopropanol and methanol, whereas dense HFMs were obtained by employing butyl acetate, ethyl acetate, hexane, toluene, water and xylene. From the aforementioned nonsolvents, a desired inner porous microstructure was achieved by using methanol, ethanol and acetone. The evaluation of the performance modifying the composition of acetone as bore fluid by adding ethanol and the effect of employing a water–acetone mixture as outer coagulant was conducted by obtaining hydrogen permeances and H_2_/CO_2_ and H_2_/N_2_ selectivities in the range of 7.6–21.0 GPU, 17.1–22.4 and 18.4–62.0, respectively, confirming the good performance of PBI HFMs (Figure 9 and Table 6).

As a consequence of the extensive research effort to obtain the best PBI HFM output, Berchtold et al. (2018), who authored the research studies of PBI HFMs analyzed here, patented a method for producing asymmetric hollow fiber membranes whose main results, taking hydrogen recovery from a syngas stream as an example, are summarized in Figure 9 and Table 6. Hydrogen permeances and H_2_/CO_2_ selectivity up to 285 GPU and 24, respectively, led these membranes to the best tradeoff and stresses the potentiality of polymeric membranes in the field of gas separation.

An additional step would consist of the addition of fillers to the PBI HFM. Considering that the selective layer thickness is normally below a micrometer, the dimension of the fillers must be smaller, with the aim of avoiding the presence of defects and particle penetration [90]. In the work developed by Yang et al. (2012), a HFM was prepared selecting Matrimid^®^ as the inner layer material and PBI/ZIF-8 as the outer layer material, co-extruded through a triple-orifice spinneret by a dry-jet/wet-quench spinning process. Material selection and disposition were considered, taking into account several factors: the brittleness of PBI membranes synthesized by a nonsolvent phase-inversion procedure that may be overcome by selecting a strong material as the inner layer; the gas transport resistance of the inner layer may be diminished by selecting a support layer material with high permeability; the employment of a polymeric material in the support layer counteracts the relative high cost of PBI/ZIF-8; the use of miscible polymers avoids the delamination phenomenon; higher permeabilities may be obtained in a single-step extrusion [38,90,99]. The employment of isopropanol in the post-treatment solvent exchange led to a better performance than methanol (Figure 9 and Table 6) [90]. The analysis of the ZIF-8 composition effect displayed maximum H_2_/CO_2_ selectivities for 10 wt.% (9.5), different to that obtained for flat sheet membranes (13.0) (Table 5 and Table 6) for 15 wt.%. The results were justified considering the intercalation of filler NPs, the defects induced during the spinning of highly concentrated solutions and the possible formation of an interface between both layers. Despite the good performance of several HFMs prepared by these authors compared to pristine PBI/Matrimid^®^ and other HFMs from the bibliography, depicted in Figure 9, the effectiveness is not as good as expected considering the addition of selective fillers. However, it should be considered that the HFM synthesis process was not the optimum process as posterior research studies showed; therefore, it is likely that better performances could be obtained from the best available synthesis method. From the comparison between single gas and H_2_:CO_2_ 50:50 experiments, competitive gas transport was confirmed, displaying a decrease in hydrogen permeance and H_2_/CO_2_ selectivity.

Villalobos et al. (2018) suggested that the incorporation of palladium into a PBI matrix for the extrusion of a HFM could enhance the inherent mechanical instability of Pd and the low permeability of PBI [111]. The nanometric size of Pd and the buffering by the polymer reduce the stress. Spinning process optimization was carried out by using Cu^2+^ that is cheaper than Pd and also leads to stable complexes with PBI imidazole groups. With the goal of providing an adequate dispersion of Pd in PBI, the reduction of Pd ions to NPs was carried out by immersing the HF in a freshly prepared NaBH_4_ solution. From TEM analysis, the agglomeration of Pd nanoparticles was confirmed, especially in the region close to the surface. Nevertheless, good H_2_/CO_2_ selectivity was reported (10) with a corresponding hydrogen permeance of 80 GPU (Figure 9 and Table 6), displaying a performance far from the optimized PBI HFM but with a good tradeoff permeance/selectivity, at least taking into account that results were obtained at low temperatures. Considering the dense layer thickness of these membranes, for the best-performance HFM (selectivity of 10), the permeability reached 176 Barrer, surpassing the Robeson upper line (2008).

Etxebarría-Benavides et al. (2020) directly worked with PBI/ZIF-8 membranes. For comparison, the optimal PBI content in dope solution was decreased for MMHFM preparation to obtain similar viscosities in the pristine PBI and PBI/ZIF-8 mixture [106]. From SEM analysis, a densified outer layer structure, a good circularity, concentricity between the inner and outer diameter and a porous substructure presenting small pores and finger-like macrovoids were attributed to pure and PBI/ZIF-8 (10 wt.%). The addition of ZIF-8 resulted in an increase in the H_2_ permeance value from 65 to 107 GPU, with H_2_/CO_2_ ideal selectivity incurring a slight decrease from 17.6 to 16.1 at 150 °C (Table 6). Hydrogen permeance and selectivity obtained for H_2_/CO_2_ 50/50 vol.% gas mixtures were lower (around 80 GPU and 13, respectively), yet higher than those obtained by Yang et al. (2012) for the same temperature and polymer/ZIF ratio, but with Matrimid^®^ as the inner layer and tested with mixed gas (37 GPU and 11.8). Consequently, the competitive sorption phenomenon can be assumed as the mixed gas permeation deviation displayed. Taking into account the preferential adsorption sites identified by simulation, for H_2_, the highest adsorption energy is located on top of the 2-methylimidazolate ring over the C=C bond (8.6 kJ/mol), and the second adsorption site corresponds to the center of the channel of the Zn (6.2 kJ/mol), whereas CO_2_ at low loading primarily occupies a site proximal to the C=C bond of 2-methylimidazolate (preferential compared to metal cluster), and at high loading occupies the site near the aperture and (at lower significance) the Zn cage center [76,114]. Therefore, the higher loading of carbon dioxide leads to its adsorption in the three sites, reducing the availability of adsorption sites and, therefore, reducing the permeances of both gases.

The features and performance of a dual layer HFM formed by a polysulfone inner layer and polyphenylsulfone (PPSU)/PBI outer layer were assessed in a recent work by Naderi et al. [108], including the influence of post-treatment procedures, such as silicone rubber coating, thermal treatment and chemical modification. XPS and FTIR analysis confirmed the ionic interaction and covalent crosslinking of PBI and PPSU, whereas SEM analysis confirmed a highly porous inner layer formed by finger-like macrovoids and an ultrathin skin layer. During permeation tests, with the introduction of and increase in PPSU composition in the outer layer, hydrogen and carbon dioxide permeation values increased, while selectivity diminished (Table 6) as a consequence of the increase in the chain–chain distance, i.e., the *d*-spacing of the outer layer polymer chains increased from 4.7 (0 wt.% PPSU) to 5.2 Å (20 wt.%). Silicone rubber coating results displayed an increase in selectivity but a permeance diminishment in the HFM without PPSU, while the HFM with PPSU did not show selectivity differences, but permeance was reduced. By the annealing procedure, ionic interaction between the sulfonic acid groups of PPSU and the imine groups of PBI was enhanced, and consequently, the selectivity increased from 4.3 (without thermal treatment) to 6.8 (120 °C), whereas hydrogen permeance remained almost constant (7.52 and 7.60 GPU, respectively), and carbon dioxide permeance decreased (from 1.71 to 1.10 GPU). Naderi et al. (2019) reported in the same study that crosslinking with 3 wt.% *α*,*α*′-dibromo-*p*-xylene resulted in a selectivity increase (7.34 compared to 6.80), but it decreased when using 6 wt.%; in both situations, hydrogen permeance dropped [108]. An increase in crosslinker loading may decrease the number of SO_3_H groups of PPSU that ionically crosslink with N sites of PBI; therefore, there is more SO_3_H group availability to interact with carbon dioxide. However, an excessive crosslinker content may cause the formation of polymer chain that is too tight. After all these treatments, considering the best-performance HFM (with PPSU, uncoated, annealed at 120 °C and crosslinked by a 3 wt.% of crosslinker), CO_2_-induced plasticization was avoided up to 20 bar. When working with binary gas mixtures of 50/50, H_2_/CO_2_ selectivities increased with permeation temperature and with the crosslinking procedure, achieving a hydrogen permeance value of 32.1 GPU and a H_2_/CO_2_ selectivity of 14.9 with a 3 wt.% crosslinker and permeation temperature of 180 °C. This value occupies a good position in the selectivity–permeability plot (Figure 9), especially taking into account that it was obtained from a gas mixture, which could be interesting for application to steam reforming and syngas stream gas separation, depending on purity requirements.

It is worth mentioning that almost all the results presented for PBI and PBI-based membranes were obtained at high temperatures, mainly considering their application in gas separation in syngas processes, which, to an extent, invalidates a correct interpretation and comparison with Matrimid^®^ and Matrimid^®^-based membrane performance, because, as explained thoroughly throughout this manuscript, temperature has a positive influence, in general, on hydrogen permeance and selectivity towards hydrogen.

Lau et al. (2010) evaluated, together with the Matrimid^®^/PBI/polysulfone dual layer HF already analyzed, the influence of the vapor phase modification of 6FDA-NDA/PES (where polyimide was synthesized from the monomers 4,4′-(hexafluoroisopropylidene) diphthalic anhydride and 1,5-napthalenediamine) dual layer HF and Torlon^®^ single layer [101]. In all of them, the imide groups are converted into amide due to the strong nucleophilicity of the ethylenediamine used in the vapor phase modification. In the 6FDA-NDA membrane, *d*-spacing (5.08 Å) reduced to 4.93 Å; in Torlon^®^, *d*-spacing was reduced from 4.35 to 4.30 Å. As occurred with PBI-Matrimid^®^/polysulfone, selectivity increased for 6FDA-NDA (from 1.69 to 34.80 or 35.52, depending on the modification method applied), although hydrogen permeance (72.59 GPU) decreased to 4.44–4.02 GPU. Nevertheless, after 1 min of treatment, Torlon^®^ displayed almost the same selectivity but reduced the permeability. Undoubtedly, 6FDA-NDA modification achieved the highest selectivities presented in this review for HFMs (Figure 9), although permeance values should be improved to obtain a good tradeoff. Considering the thickness values provided by the authors, hydrogen permeabilities achieved for modified 6FDA-NDA, Matrimid^®^/PBI and Torlon^®^ would be 20.7, 106.3 and 3.6 Barrer with selectivity values of 35.5, 7.8 and 6.4, respectively, surpassing the Robeson upper bound (2008) and even the performance of enhanced dense membranes (Figure 10).

Recently, Zhu et al. (2018) reported that the incorporation of lab-synthesized S-MIL-53 functionalized by aminosilane grafting and subsequently incorporated Ultem^®^ allowed the fabrication of an MMHFM with an increase in plasticization resistance and permeance values [113]. SEM analysis displayed that more and larger finger-like pores occurred in the outer region when increasing the S-MIL-53 content, but good filler–polymer adhesion was found. When the filler content was increased to 20 wt.%, particle agglomeration was observed, and the finger-like pores extended to the outer edge. Compared to pure Ultem^®^, the addition of 15 wt.% of MIL-53 resulted in an increase in hydrogen permeance (from 63.3 to 172.2 GPU), but a decrease in H_2_/N_2_ selectivity (from 132 to 94.1) and in H_2_/CO_2_ selectivity (from 5.2 to 4.7) (Table 6). Modified MIL-53 (S-MIL-53) provided lower hydrogen permeance (127.1 GPU) compared to the unmodified one, yet higher than the pure polymer, and higher H_2_/N_2_ selectivity (144.5), although H_2_/CO_2_ selectivity worsened (4.1), rendering these procedures unattractive for application in hydrogen recovery.

Pan et al. (2012) describe a procedure for obtaining ZIF-8 membranes supported in a ceramic yttria-stabilized zirconia HFM by a seeded growth method, which resulted in a sandwich-like structure with fingers initiating from both inner and outer sides [109]. Hydrogen permeance of 4598 GPU was reached with a H_2_/N_2_ selectivity of 11.0, H_2_/CO_2_ selectivity of 3.9 and H_2_/CH_4_ of 12.8 (Table 6). Despite the high permeation value (Figure 9) and the corresponding permeability (Figure 10), the selectivity is rather low compared to almost all the membrane performances presented in this review, although it surpasses the Robeson upper bound (2008). For binary mixtures, the competition between each gas mixture component resulted in a reduction in permeance and selectivity towards hydrogen.

## 3. Concluding Remarks

This review summarizes the past 15 years of research results of pristine Matrimid^®^ and Matrimid^®^-based materials for hydrogen recovery. It depicts a road map of the strategies developed to date, good choices and deficiencies detected. Some interesting and remarkable notes are as follows:

- Assurance of good dispersion of ZIF in the Matrimid^®^ polymer and morphology in a wide range of filler contents.
- Guarantee of the highest hydrogen recovery yield providing an adequate sweep-gas flowrate, hindering the polarization concentration phenomenon and increasing the driving force across the membrane.
- Plasticization phenomenon avoided controlling feed pressures as a consequence of the swelling effect and the polymer chain packing disruption caused by highly condensable gases, such as carbon dioxide.
- Provision of Matrimid^®^/filler kinetic diameters that facilitate the molecular sieving effect. Due to the higher condensability of CO_2_, materials hindering its solubility in the membrane are required.
- Scarcity in gas mixture research has been detected, especially considering the demonstration of the competitive sorption between hydrogen and carbon dioxide, which prevents hydrogen molecule diffusion reduction; therefore, the permeability of both gases and H_2_/CO_2_ selectivity has been compared to single gas tests.
- Performance improvement by the membrane annealing procedure, although it may affect the mechanical stability, e.g., weakening the damage tolerance.
- Improved selectivity by using sealants, although permeance values could be compromised.
- Positive correlation operating temperature–H_2_ permeability and operating temperature–H_2_/CO_2_ selectivity owing to the Arrhenius behavior in gas transport and the change from a diffusion-limited to a sorption-limited regime, respectively.
- Positive influence of ZIF addition on permeability/permeance values and selectivity towards hydrogen as a consequence of the adsorption site availability and the polymeric chain packing modification.
- Negative influence of excess ZIF on the mechanical properties of the MM/MMHF membrane.
- Solution of agglomeration and aggregation phenomena by using nanosized fillers that provide higher surface areas susceptible to being coated by the polymer.
- Improvement of hydrogen recovery by crosslinking reactions but deterioration of permeance values.
- Enhancement of H_2_/CO_2_ selectivity in HFMs but poorer results in the separation of hydrogen from N_2_, CH_4_ and CO.
- Importance of operating parameters in HFM preparation in the final performance: draw rate, dopes solution, coagulation bath, solvent exchange and post-treatment.

The good behavior of Matrimid^®^ and Matrimid^®^-based membranes is proven, but further improvements and a thorough assessment of real streams are still challenges to be resolved. This will be easily faced by exploiting the computational tools that enable the screening of the polymeric material and the filler that better fit with the purpose and the mechanisms involved in the gas transport process [76,115].

## Figures and Tables

**Table 1:** Main features of membrane technology for H_2_ recovery.

Membrane Material	Strengths	Weaknesses
Metallic	Mechanical durability; resistance to H_2_ embrittlement; selectivity (dense)	H_2_ fluxes; chemical and thermal stability; cost and H_2_ embrittlement at low pressure and temperature in some materials, such as Pd
Silica	Tunable nature; high-temperature and high-pressure stability of microporous silica; high surface area; resistance to H_2_ embrittlement	Cost; manufacture reproducibility; stability at high temperature and embrittlement
Zeolite	Chemical, mechanical and thermal stability	Cost; manufacture reproducibility
Carbon-based	Versatility	Cost; selectivity; brittleness; chemical, mechanical and thermal stability
Polymer	Diffusivity; selectivity; H_2_ fluxes; permeabilities; cost and processability	Chemical, mechanical and thermal stability

**Table 3:** Main bibliographical data in terms of pure gas H_2_, N_2_, CO_2_, CH_4_ and CO permeance (Pe) and selectivities through Matrimid^®^ HFMs.

Ref.	*T* (°C)	Δ*P* (Bar)	*P*e_H_2__ (GPU)	*P*e_N_2__ (GPU)	*P*e_CO_2__ (GPU)	*P*e_CH__4_ (GPU)	*P*e_CO_ (GPU)	α_H_2_/N_2__	α_H_2_/CO_2__	α_H_2_/CH_4__	α_H_2_/CO_	Comments on Membrane Fabrication
Bernardo (2019) [74]	25	1.0	40.0	0.53	13.3	0.44		75.5	3.0	90.9		M1: Shell fluid none. Dope flow rate 5 g min^−1^ Protocol 1: no solvent-exchange
			47.0	0.63	21.9	0.54		74.6	2.1	87.0		M2: Shell fluid NMP/water. Dope flow rate 5 g min^−1^ Protocol 1
			41.6	0.58	17.0	0.52		71.7	2.4	80.0		M3: Shell fluid NMP/water. Dope flow rate 3.6 g min^−1^ Protocol 1
David (2012) [68]	30	2.3	66.7	0.91	13.4		1.6	73.3	5.0		41.7	air gap 12 cm
		4.1	65.8	0.94	14.4		1.6	70.0	4.6		41.1	
		6.1	66.9	0.93	15.9		1.7	71.9	4.2		39.4	
		8.0	64.1	0.92	16.8		1.6	69.7	3.8		40.1	
		10.0	65.5	0.93	19.1		1.7	70.4	3.4		38.5	
		2.2	159.0	9.1	40		9.1	17.5	4.0		17.5	air gap 3 cm
		4.1	164.2	10.0	41.0		9.5	16.4	4.0		17.3	
		6.1	169.6	10.3	43.0		10.1	16.5	3.9		16.8	
Favvas (2007) [72]	40		342.2	23.1	84.9	30.6	26.0	14.8	4.0	11.2	13.2	Without pyrolysis
	40		2.9	0.11	0.08	0.09	0.07	26.1	37.8	31.9	43.5	M1, N_2_ atmosphere
			20.5	0.27	6.3	0.30	0.59	75.8	3.2	68.2	34.7	M2, H_2_O atmosphere
			17.6	0.12	2.6	0.16	0.33	146.5	6.7	109.9	53.3	M3, CO_2_ atmosphere
	60		4.1	0.05	0.15	0.06	0.09	81.6	27.2	68.0	45.3	M1
			27.6	0.54	9.9	0.44	1.00	51.1	2.8	62.4	27.6	M2
			26.1	0.27	4.0	0.19	0.48	96.7	6.5	137.4	54.4	M3
	100		7.8	0.08	0.28	0.08	0.16	101.6	27.9	97.8	48.9	M1
			53.3	1.22	17.3	1.91	2.08	43.7	3.1	27.9	25.6	M2
			36.5	0.57	6.8	0.37	0.85	64.0	5.4	98.6	42.9	M3
Peer (2007) [73]	20	9.0	70.2				4.1				17.0	UBE polyimide
	40	9.0	74.0				4.3				17.3	
	60	9.0	76.7				3.7				21.0	
	80	9.0	80.7				2.2				37.0	

## Data Availability

Not applicable.

## Abbreviations

AFM	atomic force microscopy
APTES	3-aminopropyltriethoxysilane
DCM	dichloromethane
DMA	dynamic mechanical analyzer
DMAc	N,N-dimethylacetamide
DMF	dimethyl formamide
DSC	differential scanning calorimetry
EDX	energy dispersive X-ray
6FDA	4,4′-(hexafluoroisopropylidene) diphthalic anhydride
FFV	fractional free volume
FTIR	Fourier transform infrared spectroscopy
GBL	gamma-butyrolactone
HFM	hollow fiber membrane
MMHFM	hollow fiber mixed matrix membrane
MMM	mixed matrix membrane
MOF	metal organic framework
NDA	1,5-napthalenediamine
NP	nanoparticle
NMP	N-methylpyrrolidone
NMR	nuclear magnetic resonance
PALS	positron annihilation lifetime spectroscopy
PBI	polybenzimidazole
PDA	polydopamine
PEI	polyetherimide
PES	polyethersulfone
PMDA	pyromellitic dianhydride
PPSU	polyphenylsulfone
PSA	pressure swing adsorption
SEM	scanning electron microscopy
TCE	1,1,2,2-tetrachloroethane
TEM	transmission electron microscopy
TGA	thermogravimetric analysis
THF	tetrahydrofuran
XPS	X-ray photoelectron spectroscopy
XRD	X-ray diffraction
ZIF	zeolitic imidazolate framework
